# Supplementary figures and images for: Continuous endosomes form functional subdomains and orchestrate rapid membrane trafficking in trypanosomes
Source: eLife. 2024 Apr 15;12:RP91194. doi: 10.7554/eLife.91194 (PMC11018342; doi:10.7554/eLife.91194)

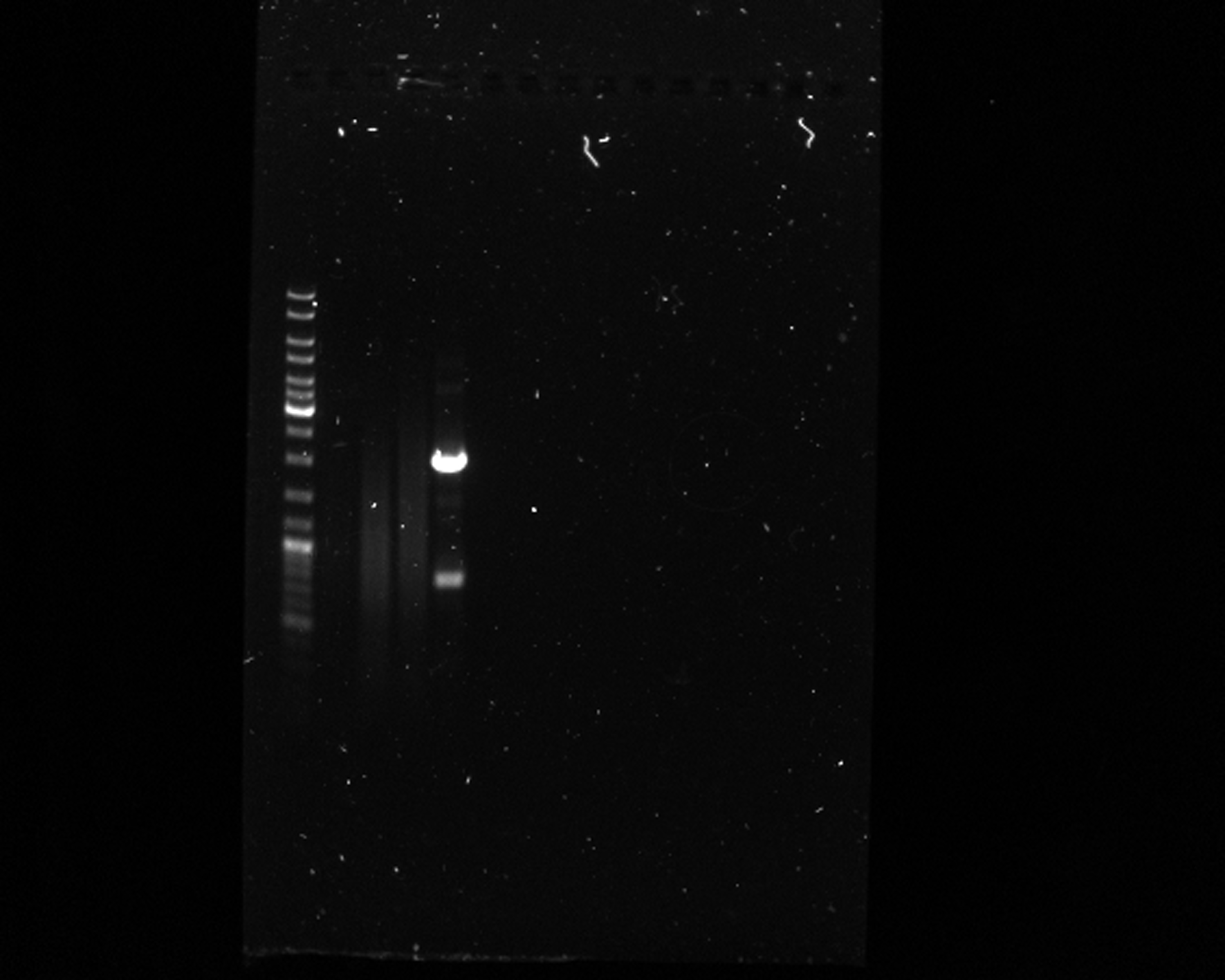

Supplement: Figure 2—figure supplement 2—source data 1. [file elife-91194-fig2-figsupp2-data1.zip › 2023_05_10_124431Gel Of PCR of gDNA EP1Halo.tif]

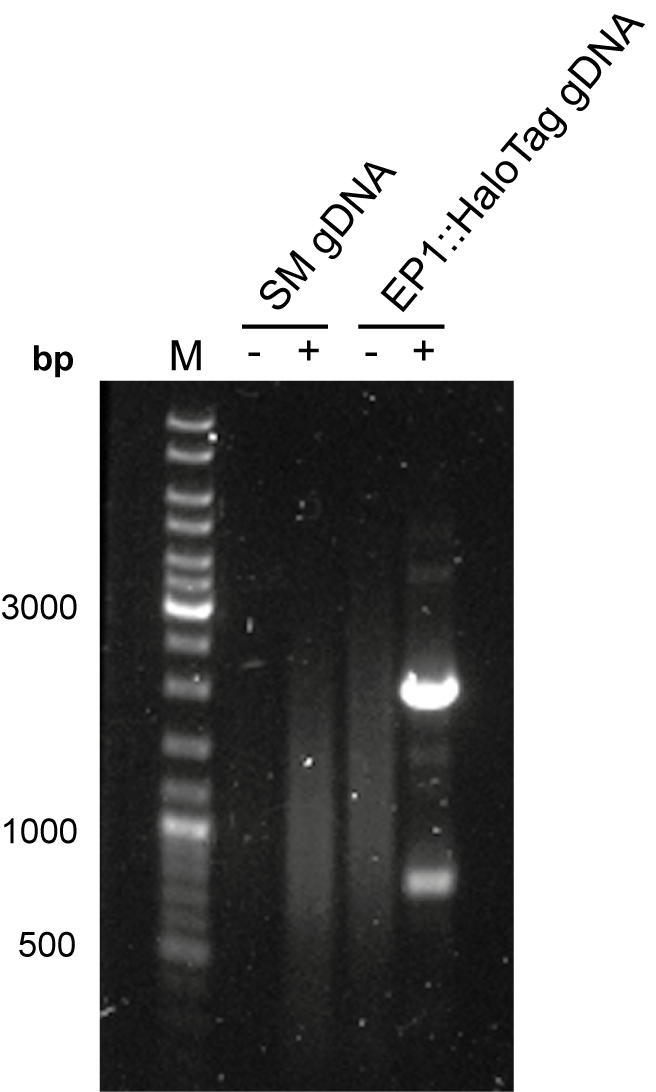

Supplement: Figure 2—figure supplement 2—source data 2. [file elife-91194-fig2-figsupp2-data2.zip › Gel_labelled.png]

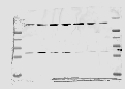

Supplement: Figure 3—figure supplement 1—source data 1. [file elife-91194-fig3-figsupp1-data1.zip › 0003350_02/0003350_02_TH.jpg]

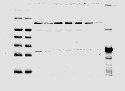

Supplement: Figure 3—figure supplement 1—source data 1. [file elife-91194-fig3-figsupp1-data1.zip › 0003349_02/0003349_02_TH.jpg]

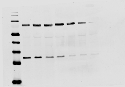

Supplement: Figure 3—figure supplement 1—source data 1. [file elife-91194-fig3-figsupp1-data1.zip › 0003348_02/0003348_02_TH.jpg]

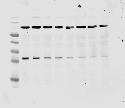

Supplement: Figure 3—figure supplement 1—source data 1. [file elife-91194-fig3-figsupp1-data1.zip › 0003230_02/0003230_02_TH.jpg]

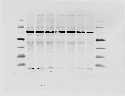

Supplement: Figure 3—figure supplement 1—source data 1. [file elife-91194-fig3-figsupp1-data1.zip › 0003074_02/0003074_02_TH.jpg]

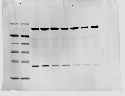

Supplement: Figure 3—figure supplement 1—source data 1. [file elife-91194-fig3-figsupp1-data1.zip › 0003036_02/0003036_02_TH.jpg]

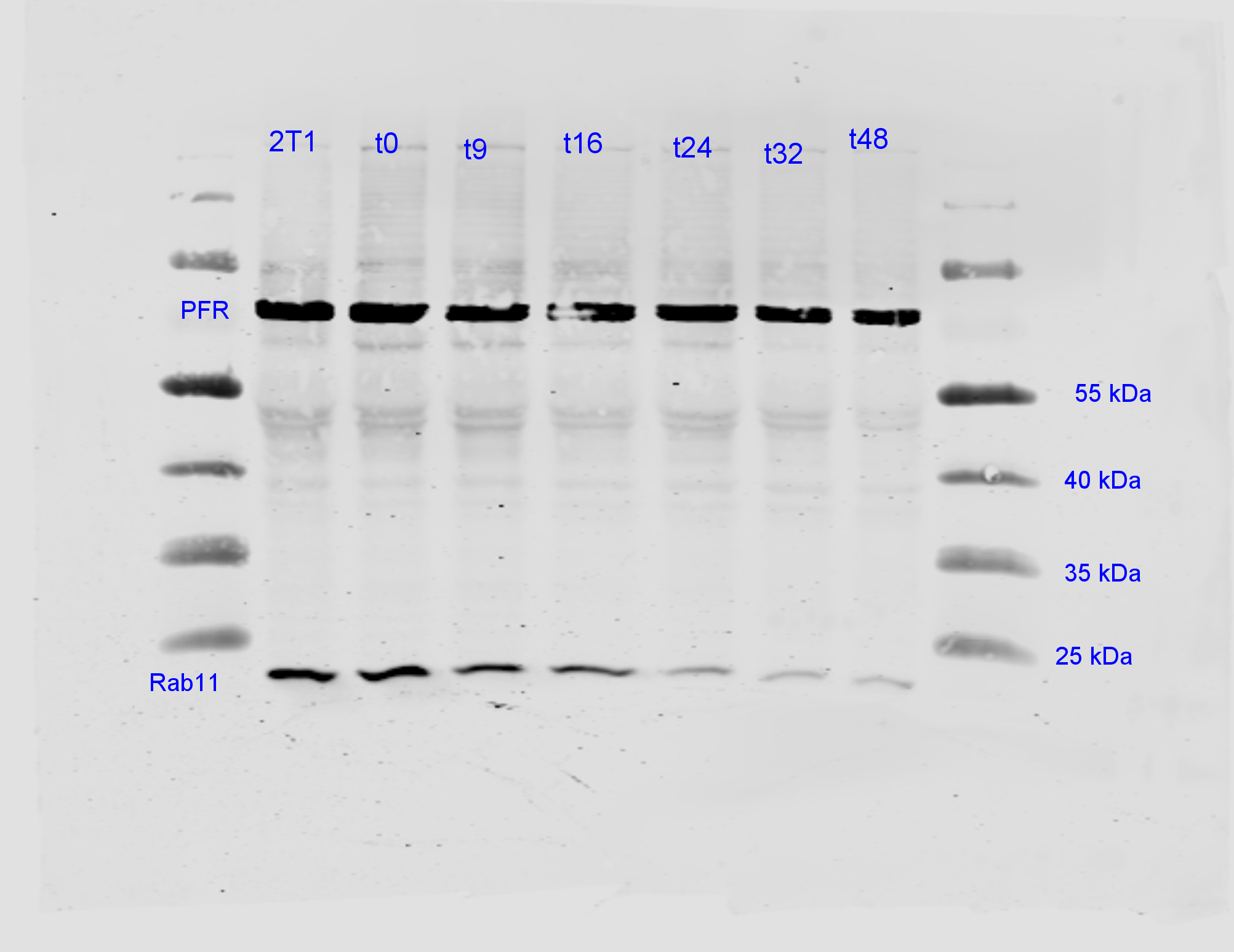

Supplement: Figure 3—figure supplement 1—source data 2. [file elife-91194-fig3-figsupp1-data2.zip › anti-Rab11 rb.png]

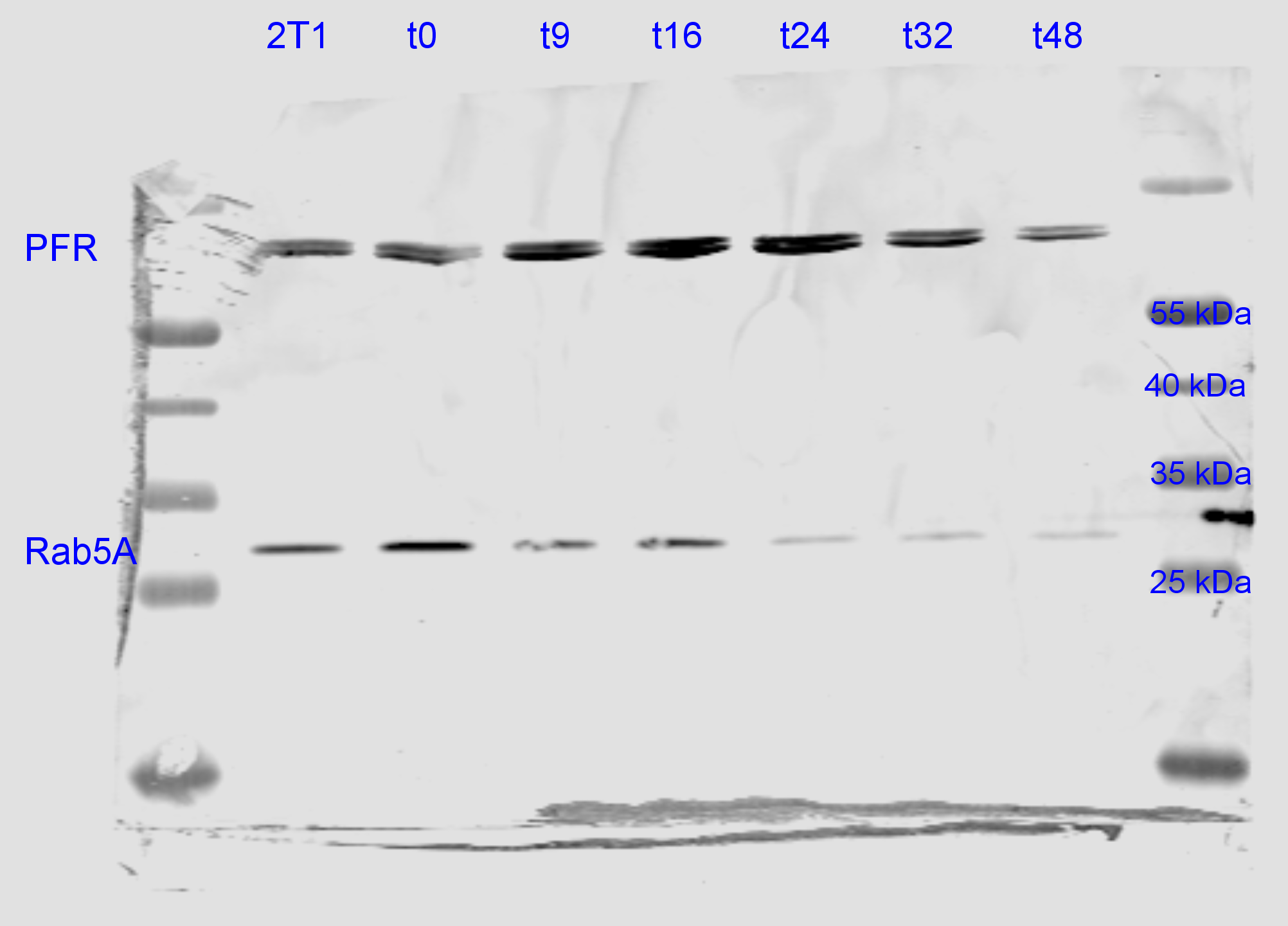

Supplement: Figure 3—figure supplement 1—source data 2. [file elife-91194-fig3-figsupp1-data2.zip › anti-Rab5A rat2.png]

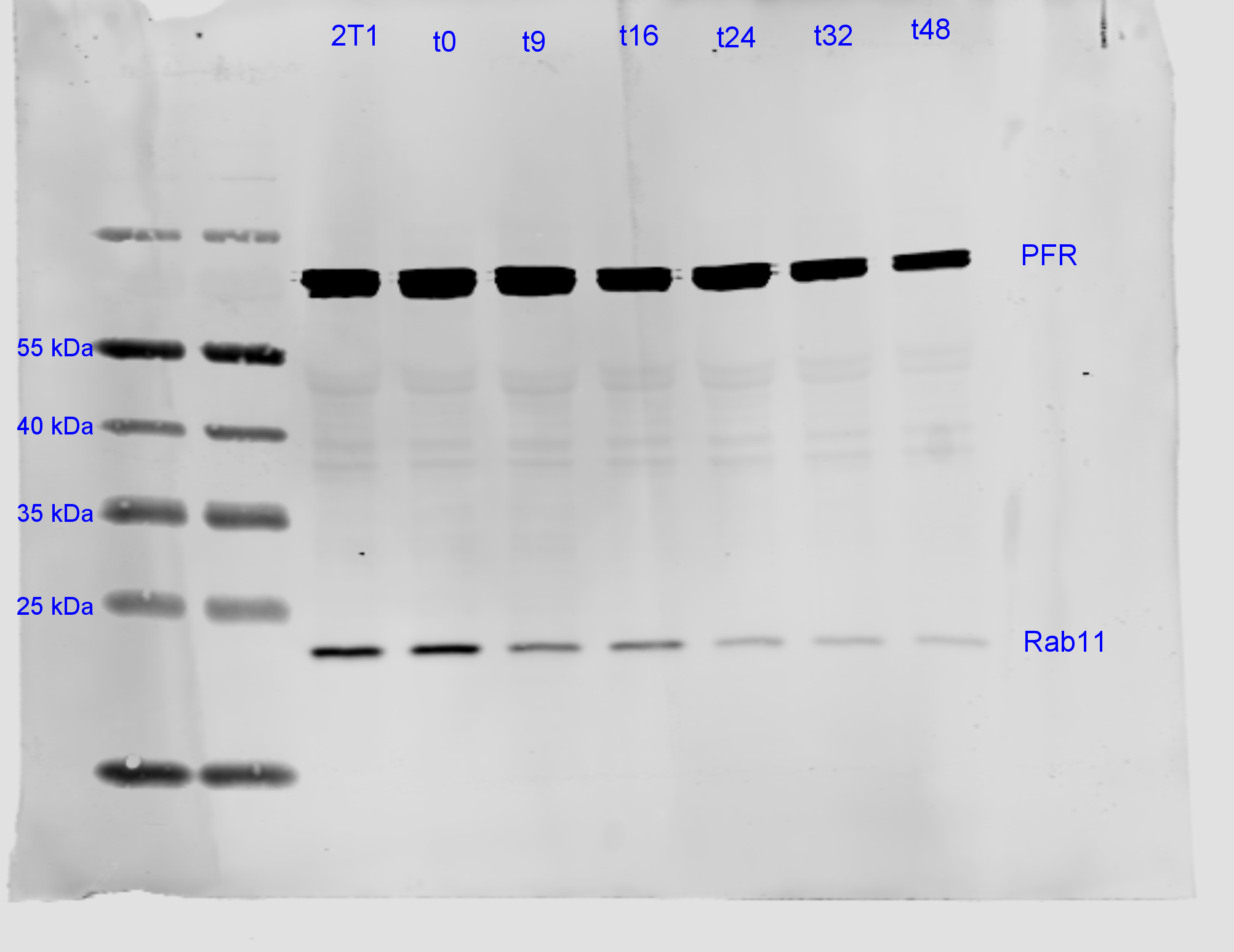

Supplement: Figure 3—figure supplement 1—source data 2. [file elife-91194-fig3-figsupp1-data2.zip › anti-Rab11 gp.png]

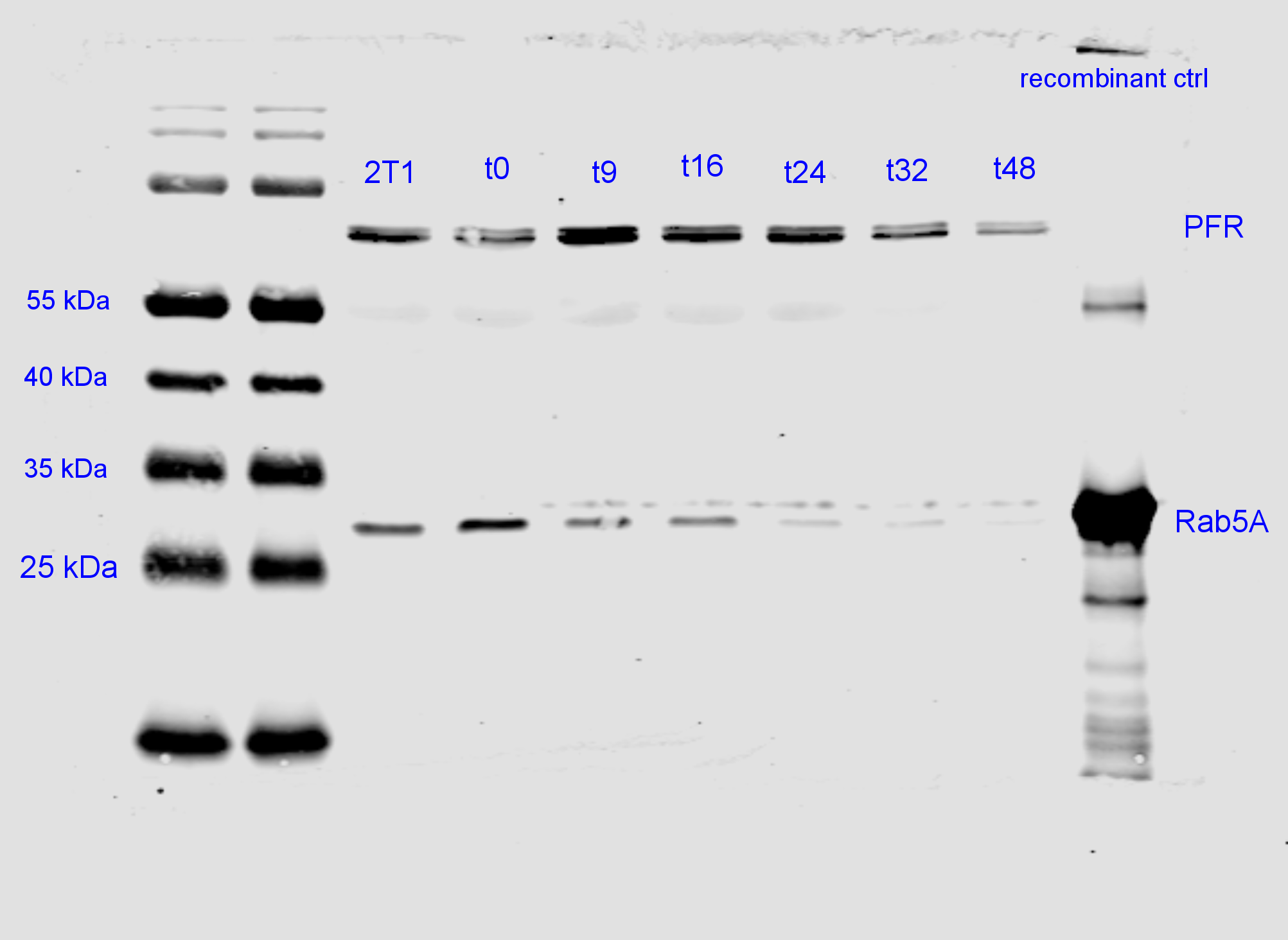

Supplement: Figure 3—figure supplement 1—source data 2. [file elife-91194-fig3-figsupp1-data2.zip › anti-Rab5A rat1.png]

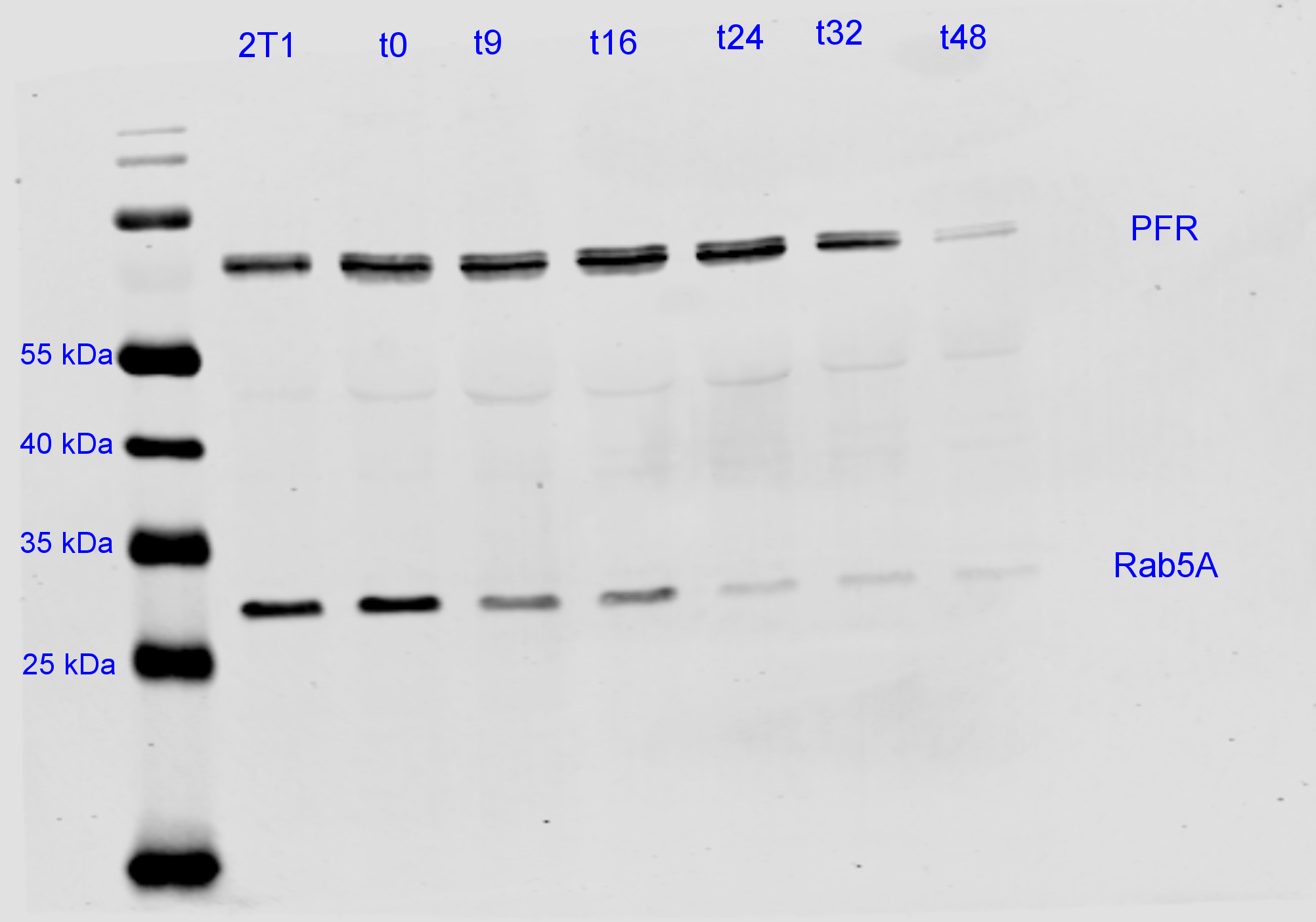

Supplement: Figure 3—figure supplement 1—source data 2. [file elife-91194-fig3-figsupp1-data2.zip › anti-Rab5A rb.png]

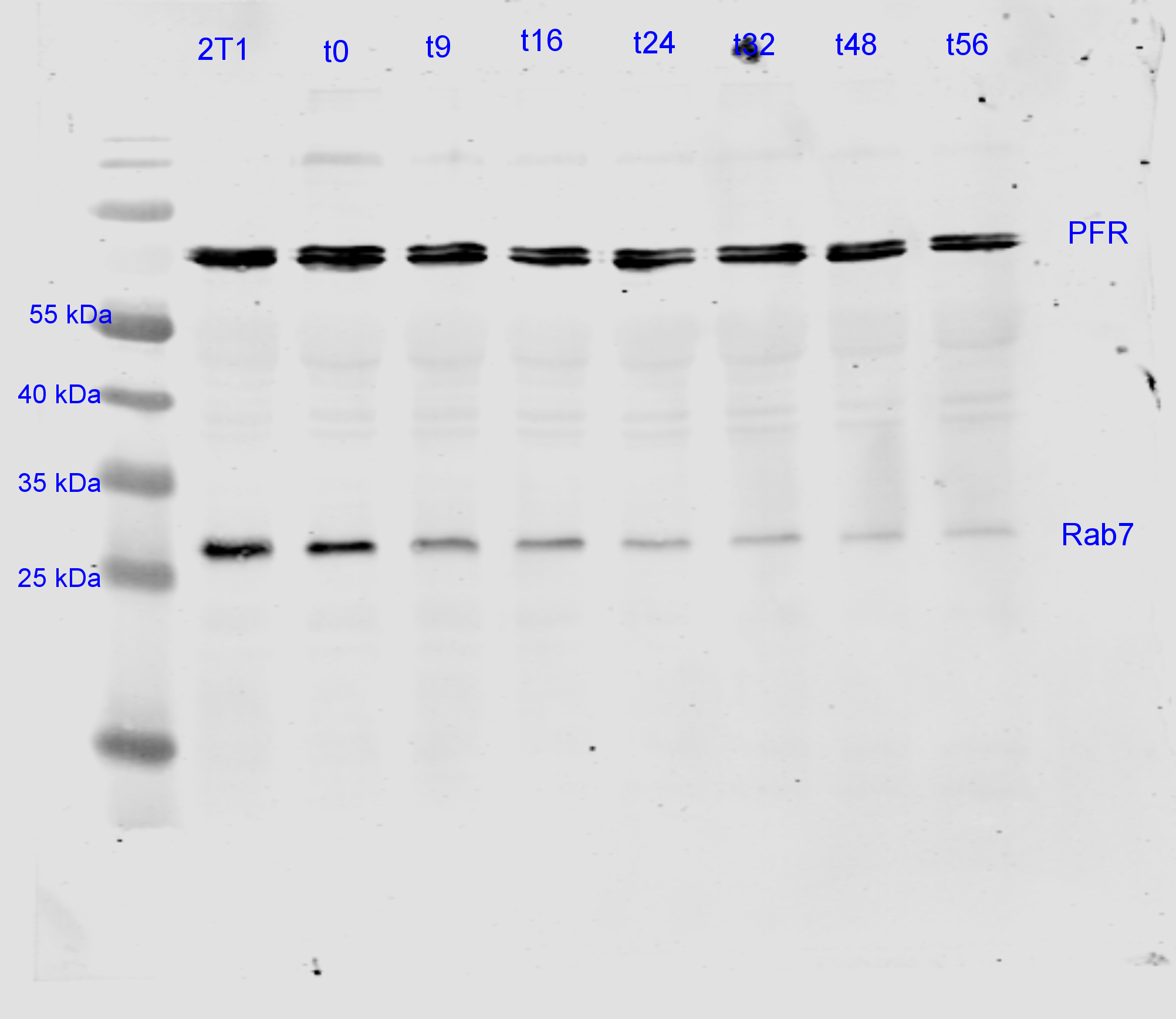

Supplement: Figure 3—figure supplement 1—source data 2. [file elife-91194-fig3-figsupp1-data2.zip › anti-Rab7 gp.png]

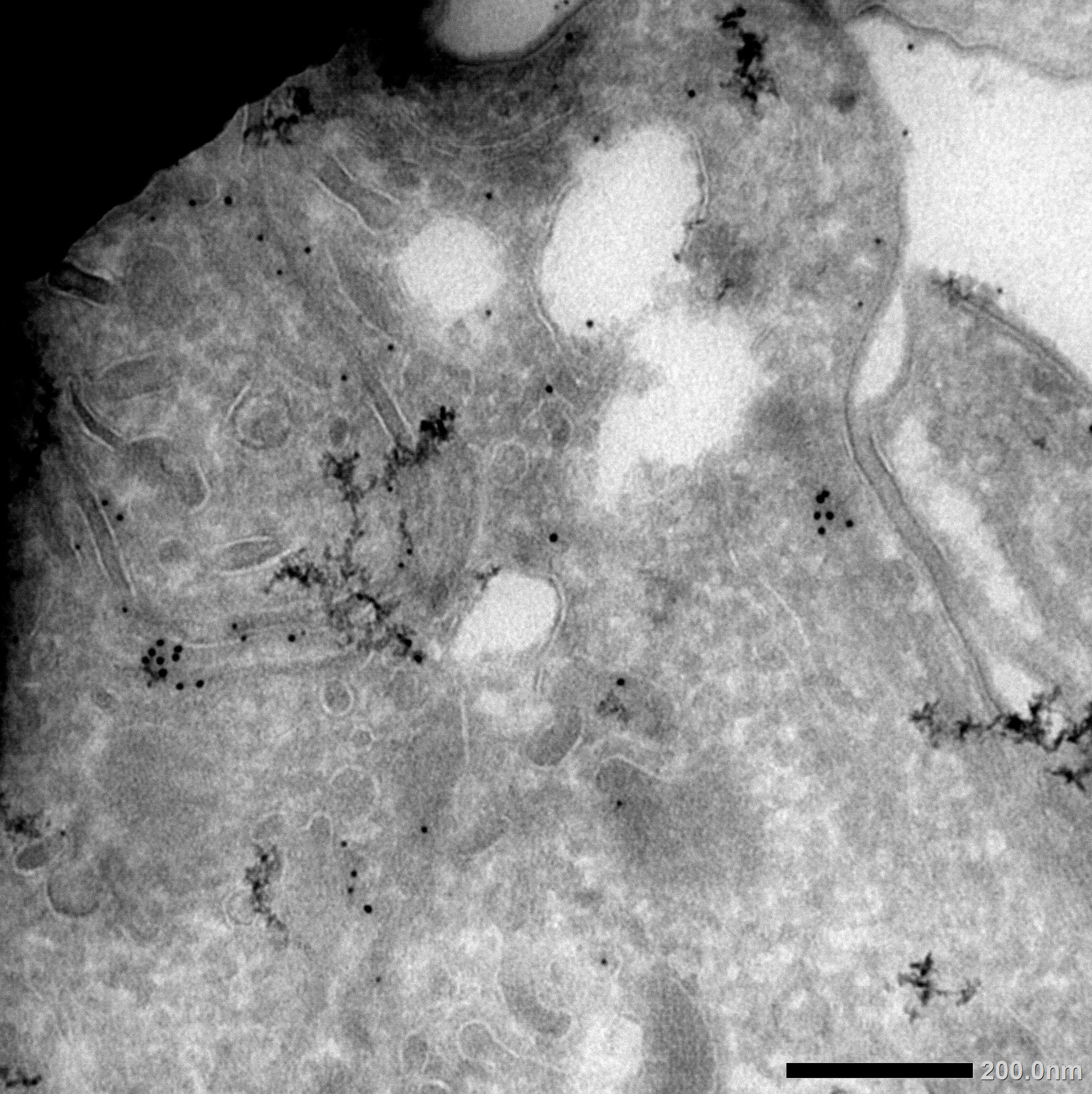

Supplement: Figure 4—source data 1. [file elife-91194-fig4-data1.zip › F.jpg]

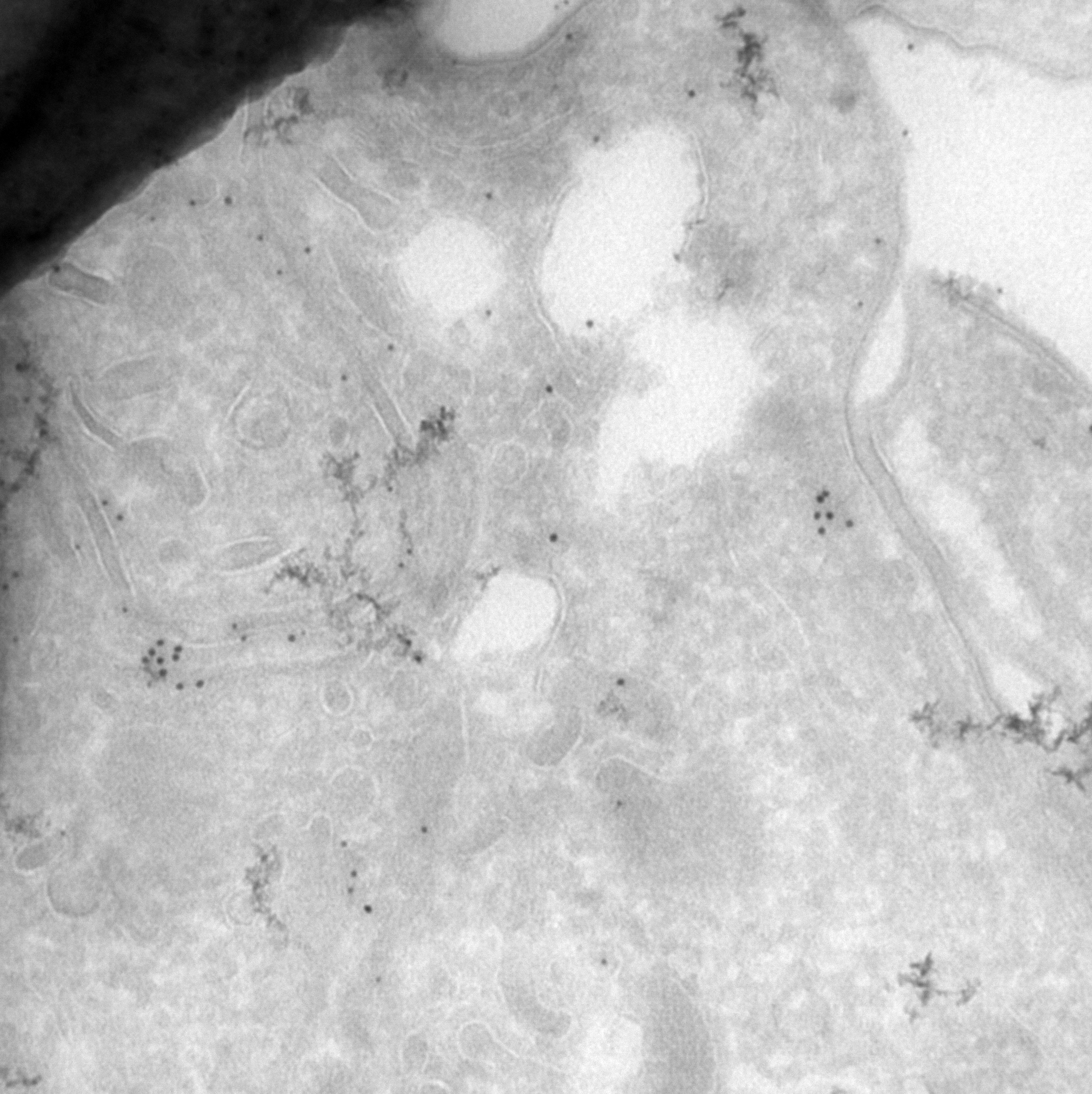

Supplement: Figure 4—source data 1. [file elife-91194-fig4-data1.zip › F.tif]

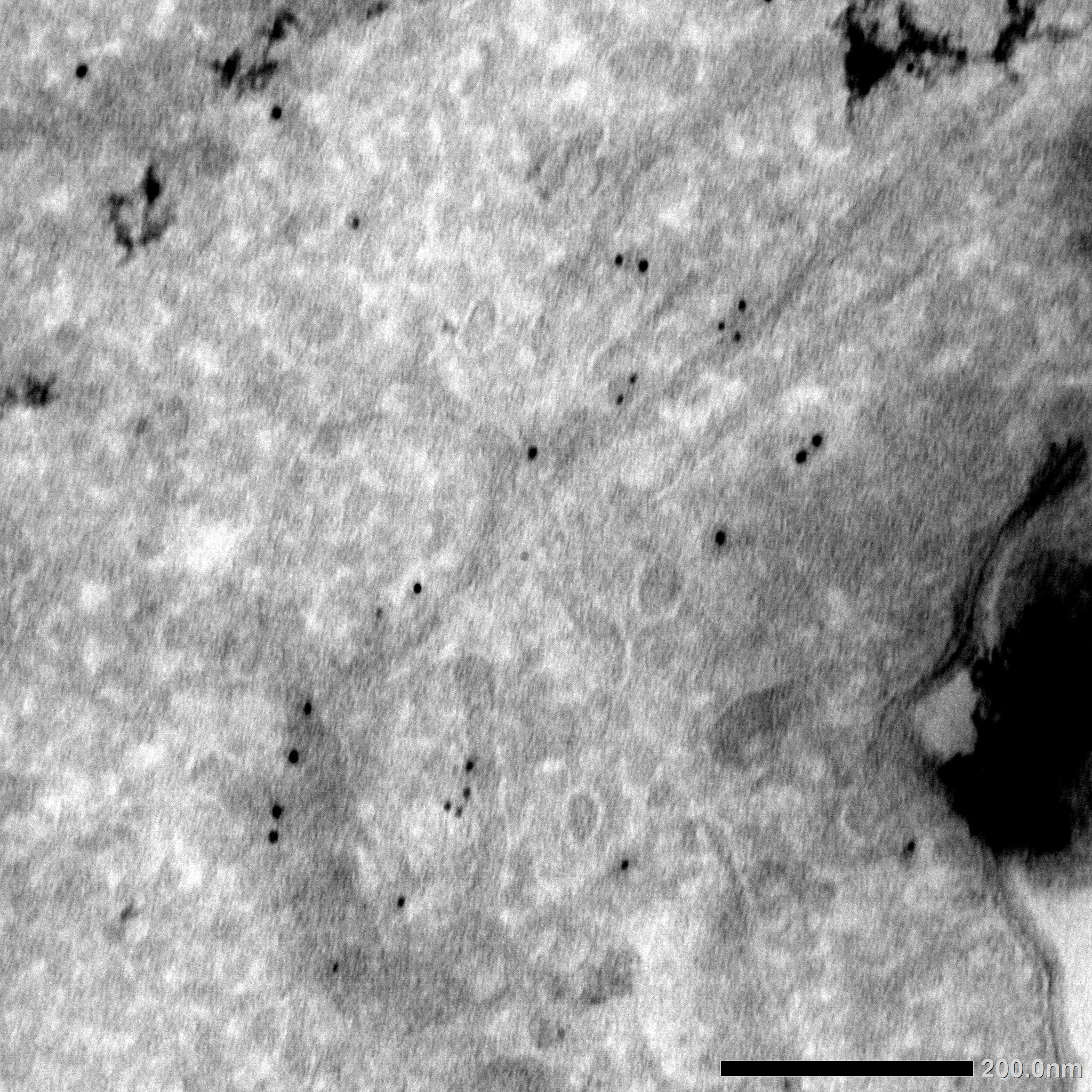

Supplement: Figure 4—source data 1. [file elife-91194-fig4-data1.zip › G.jpg]

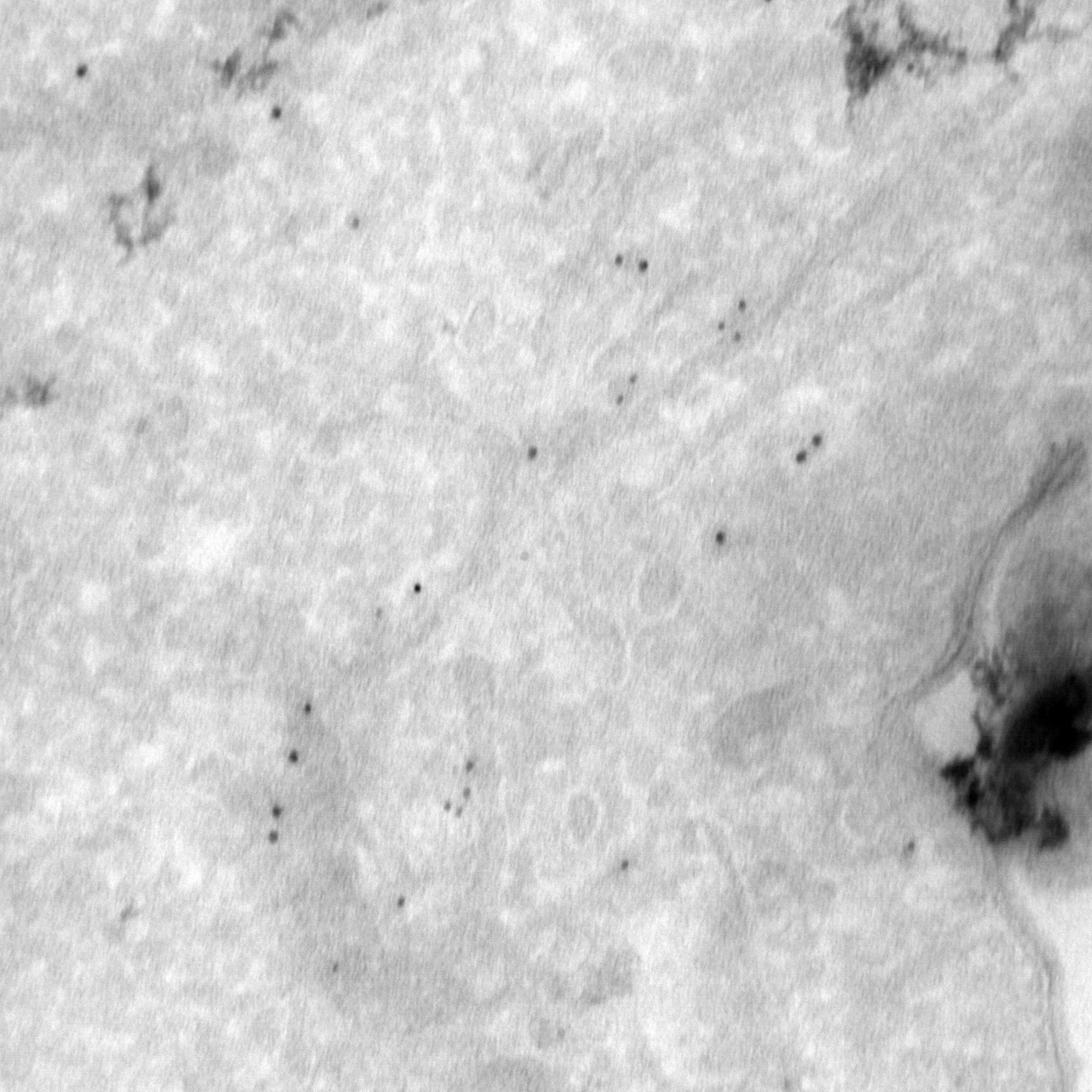

Supplement: Figure 4—source data 1. [file elife-91194-fig4-data1.zip › G.tif]

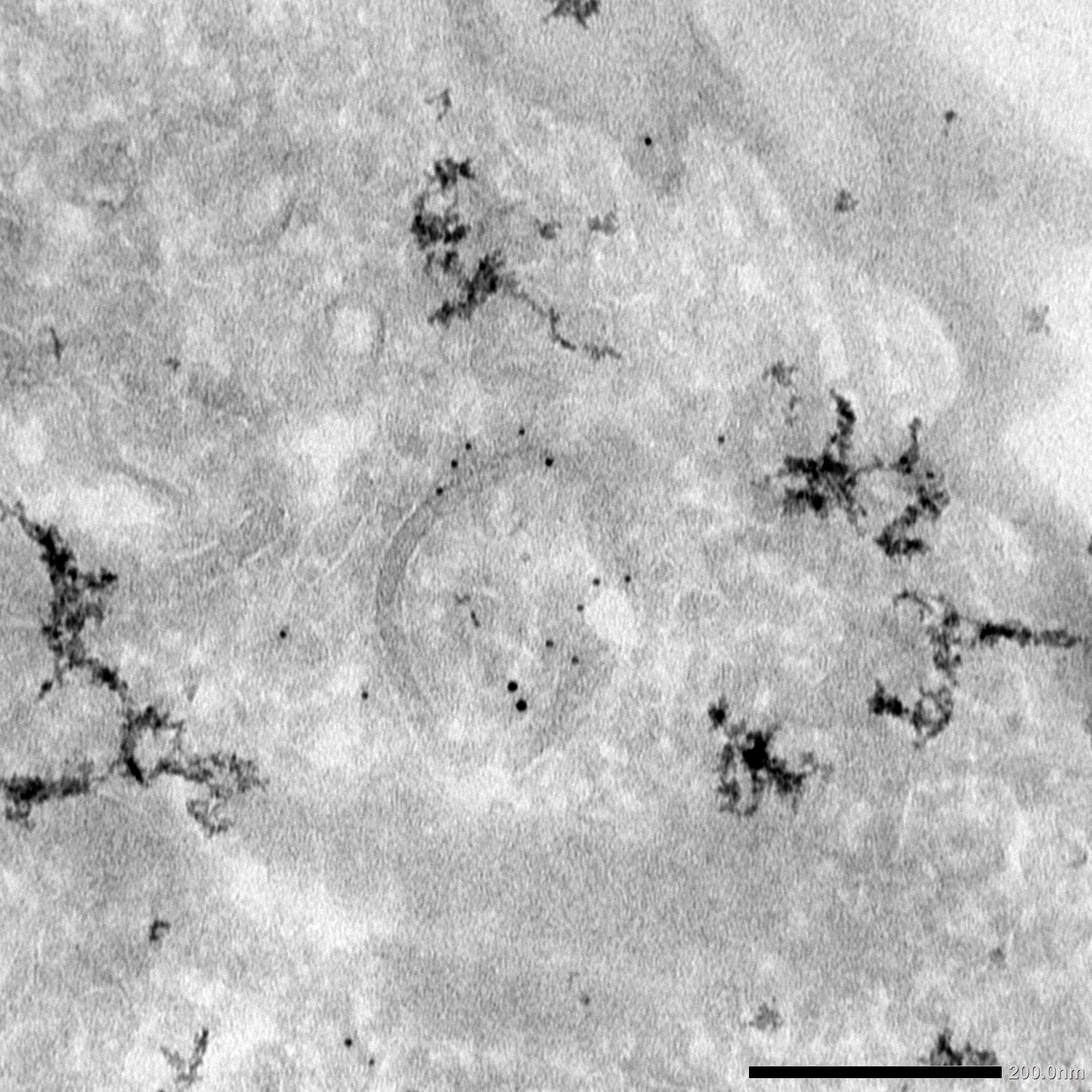

Supplement: Figure 4—source data 1. [file elife-91194-fig4-data1.zip › B.jpg]

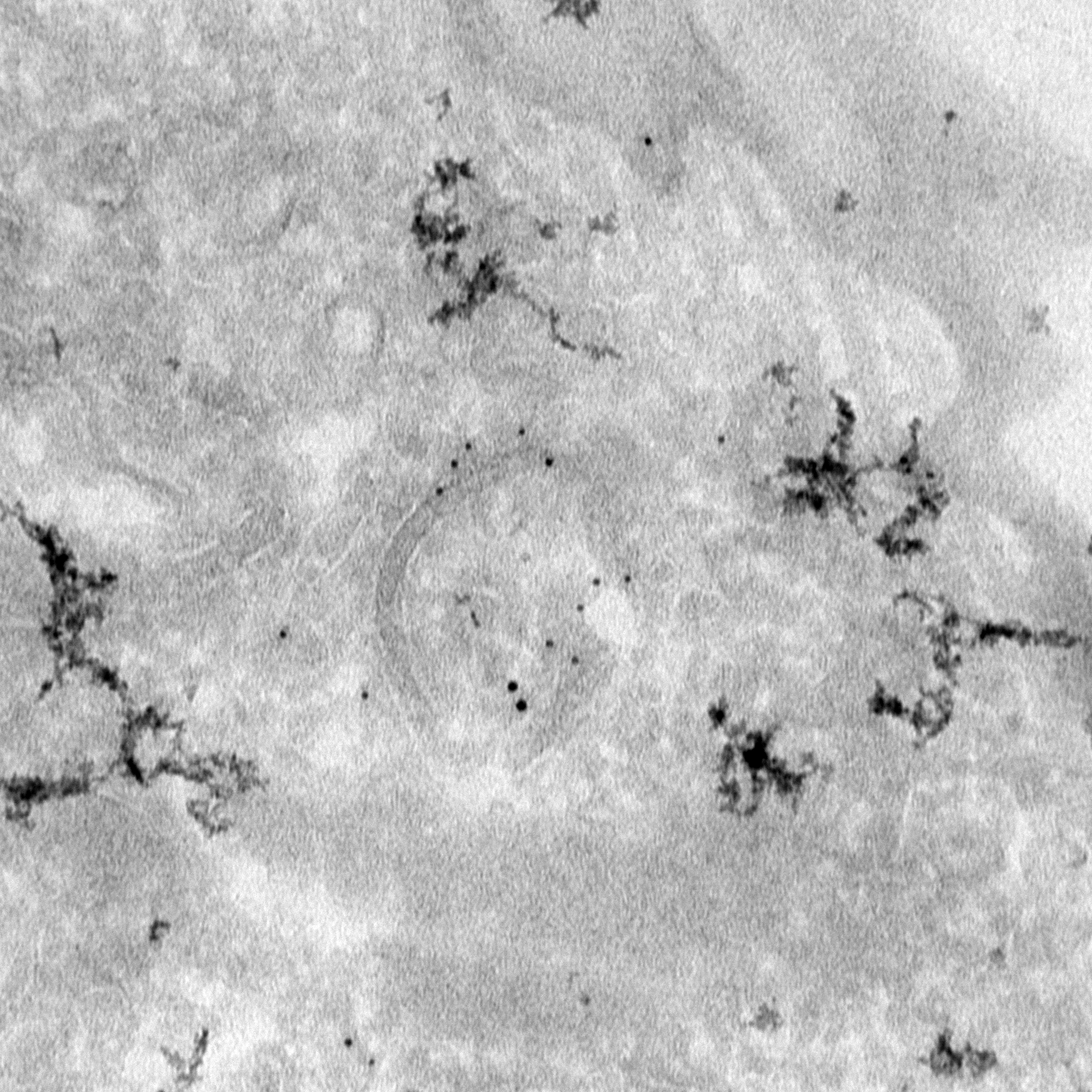

Supplement: Figure 4—source data 1. [file elife-91194-fig4-data1.zip › B.tif]

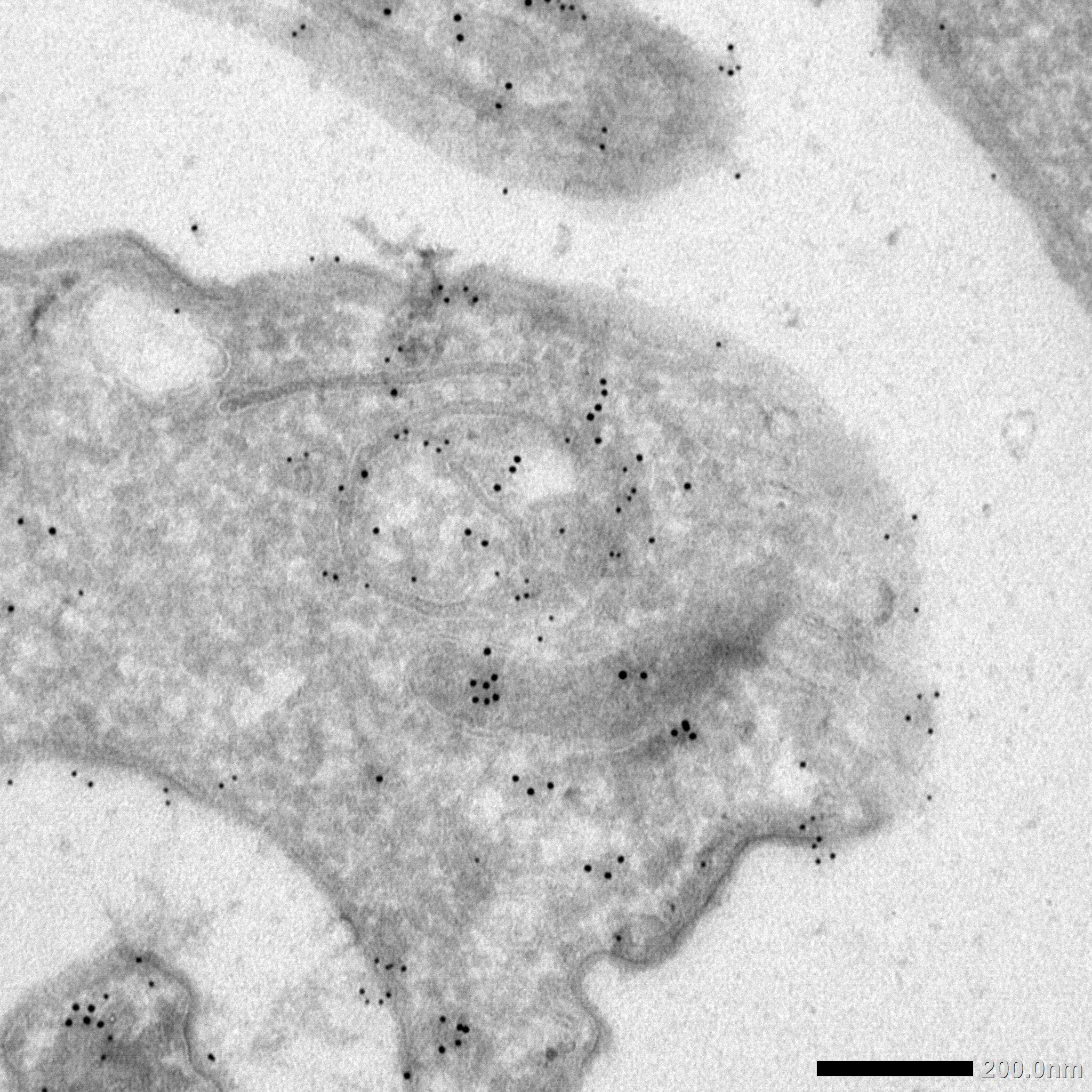

Supplement: Figure 4—source data 1. [file elife-91194-fig4-data1.zip › C.jpg]

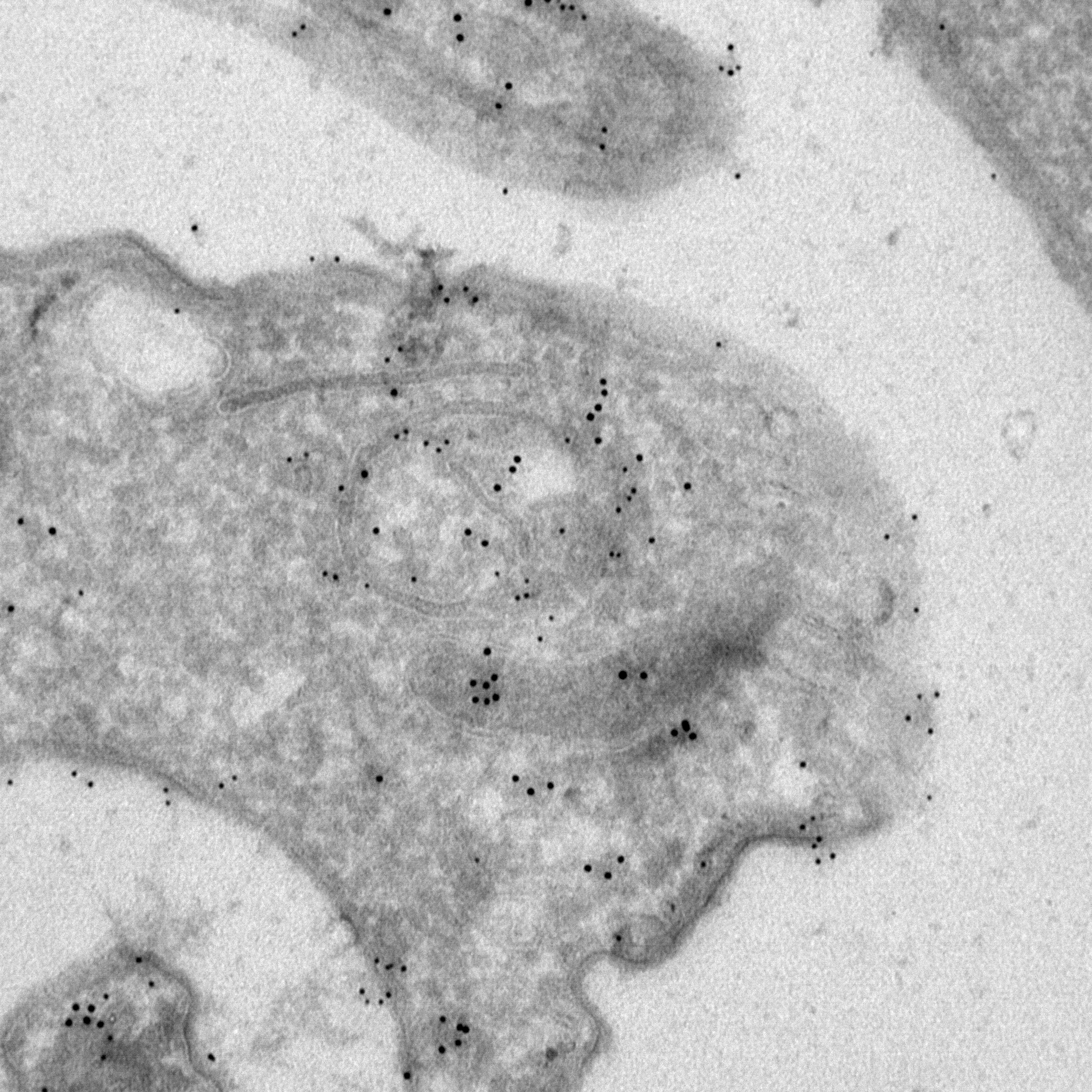

Supplement: Figure 4—source data 1. [file elife-91194-fig4-data1.zip › C.tif]

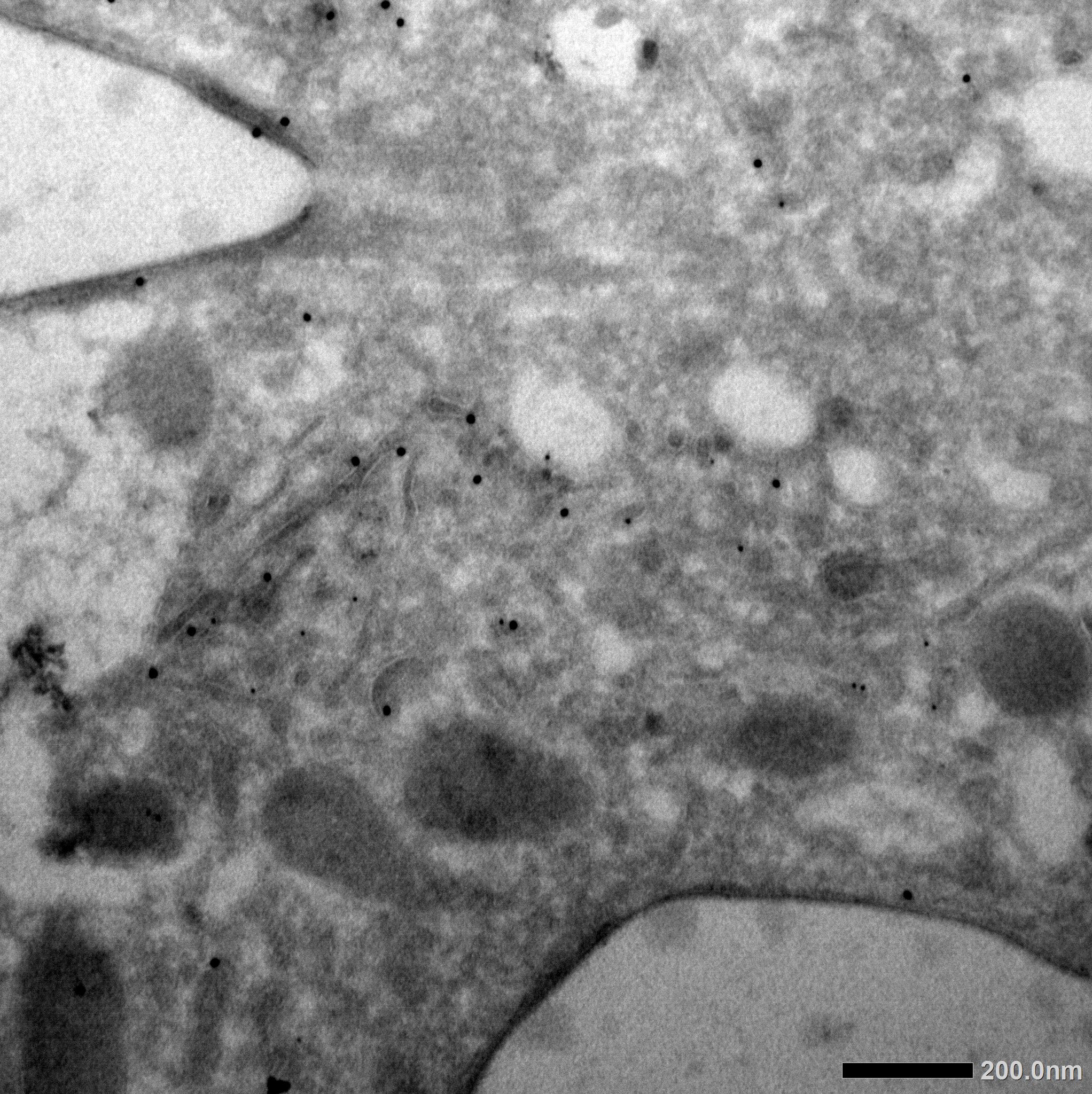

Supplement: Figure 4—source data 1. [file elife-91194-fig4-data1.zip › D.jpg]

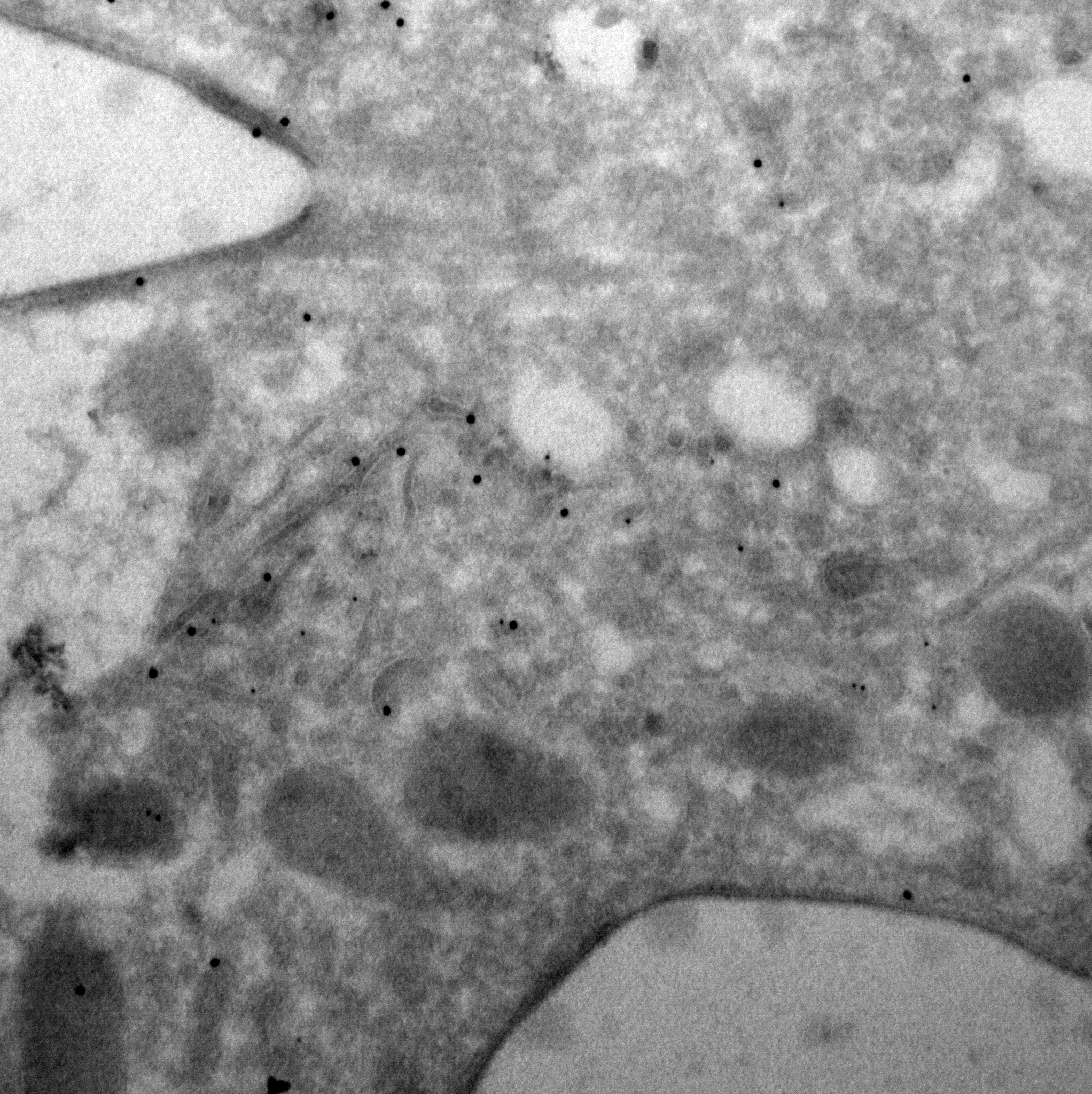

Supplement: Figure 4—source data 1. [file elife-91194-fig4-data1.zip › D.tif]

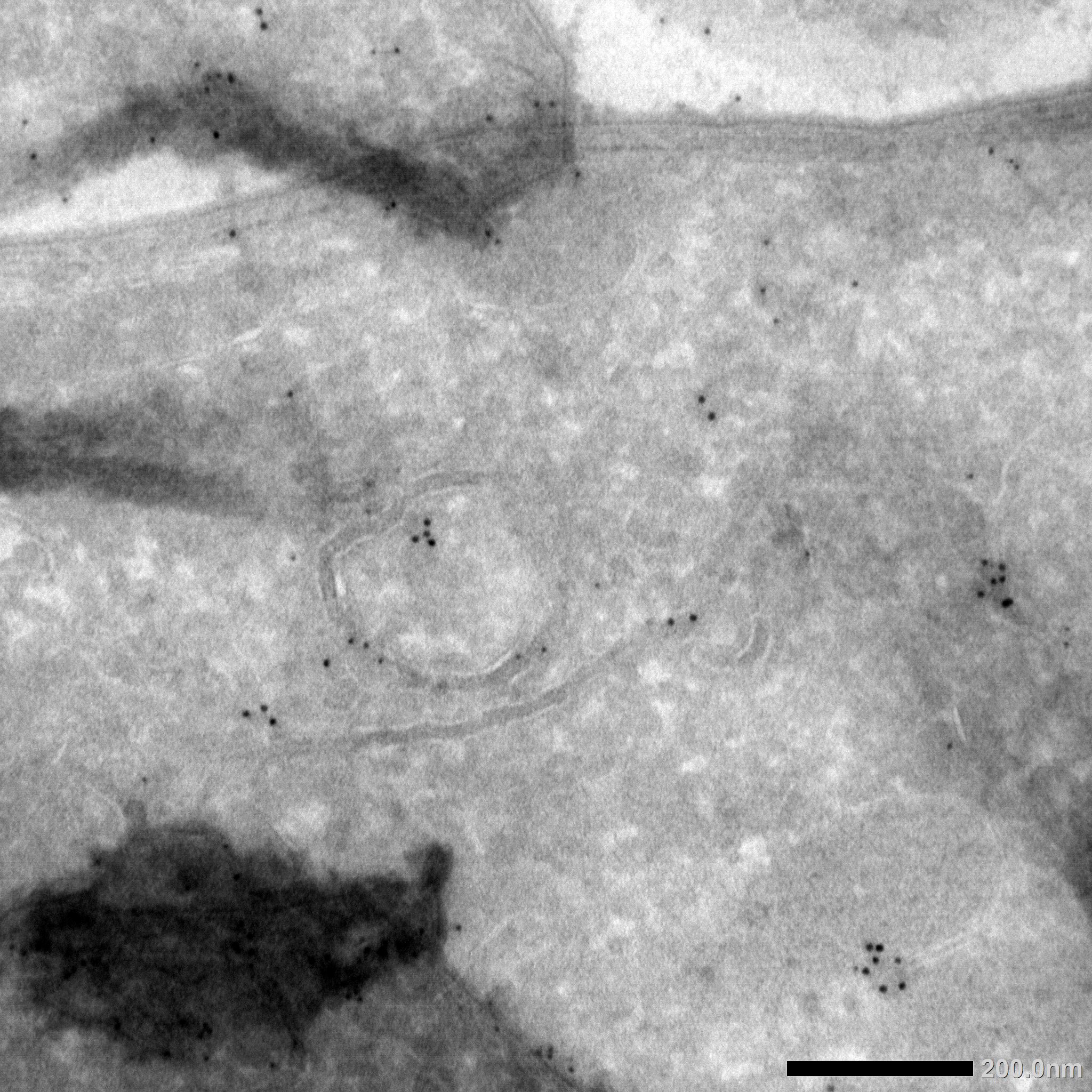

Supplement: Figure 4—source data 1. [file elife-91194-fig4-data1.zip › E.jpg]

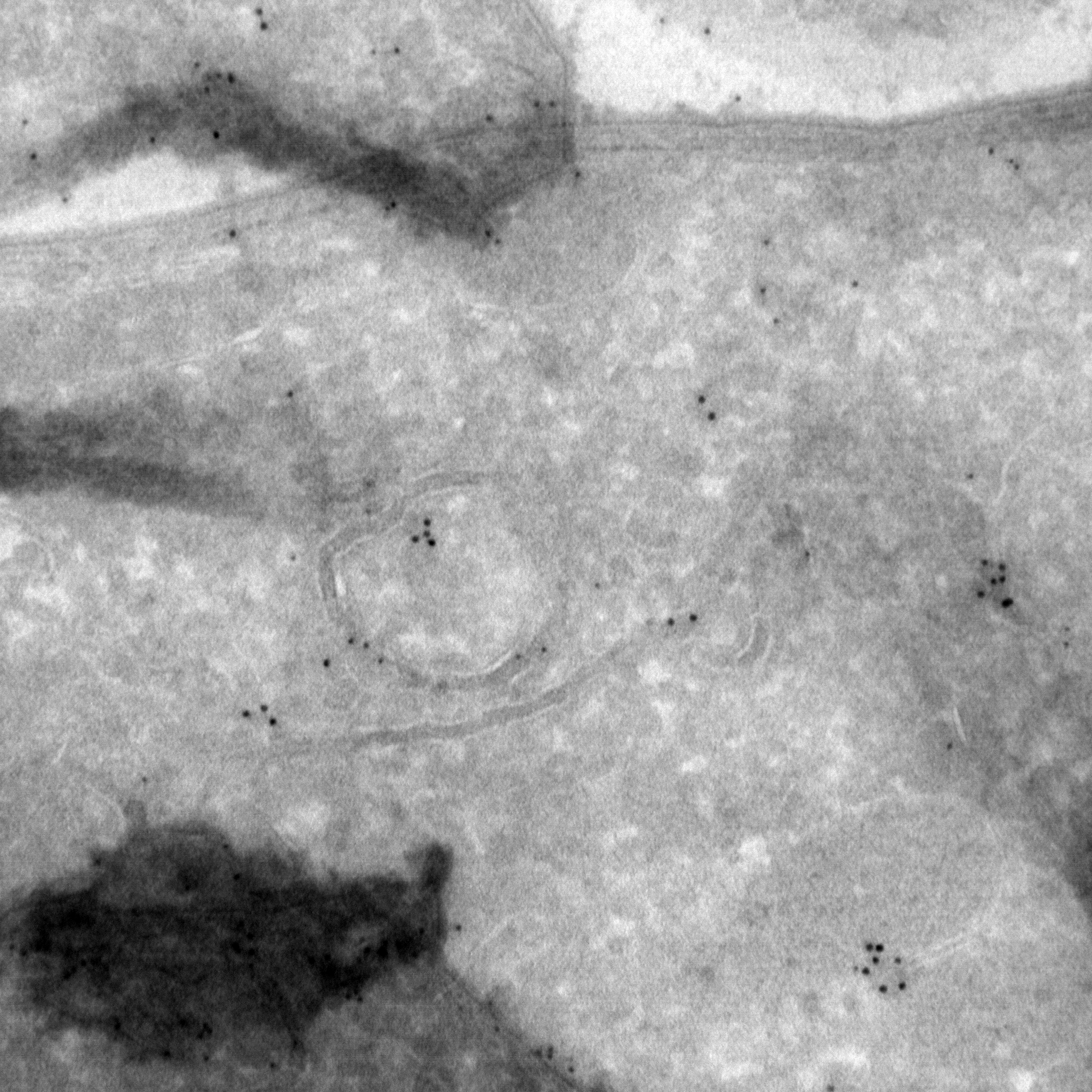

Supplement: Figure 4—source data 1. [file elife-91194-fig4-data1.zip › E.tif]

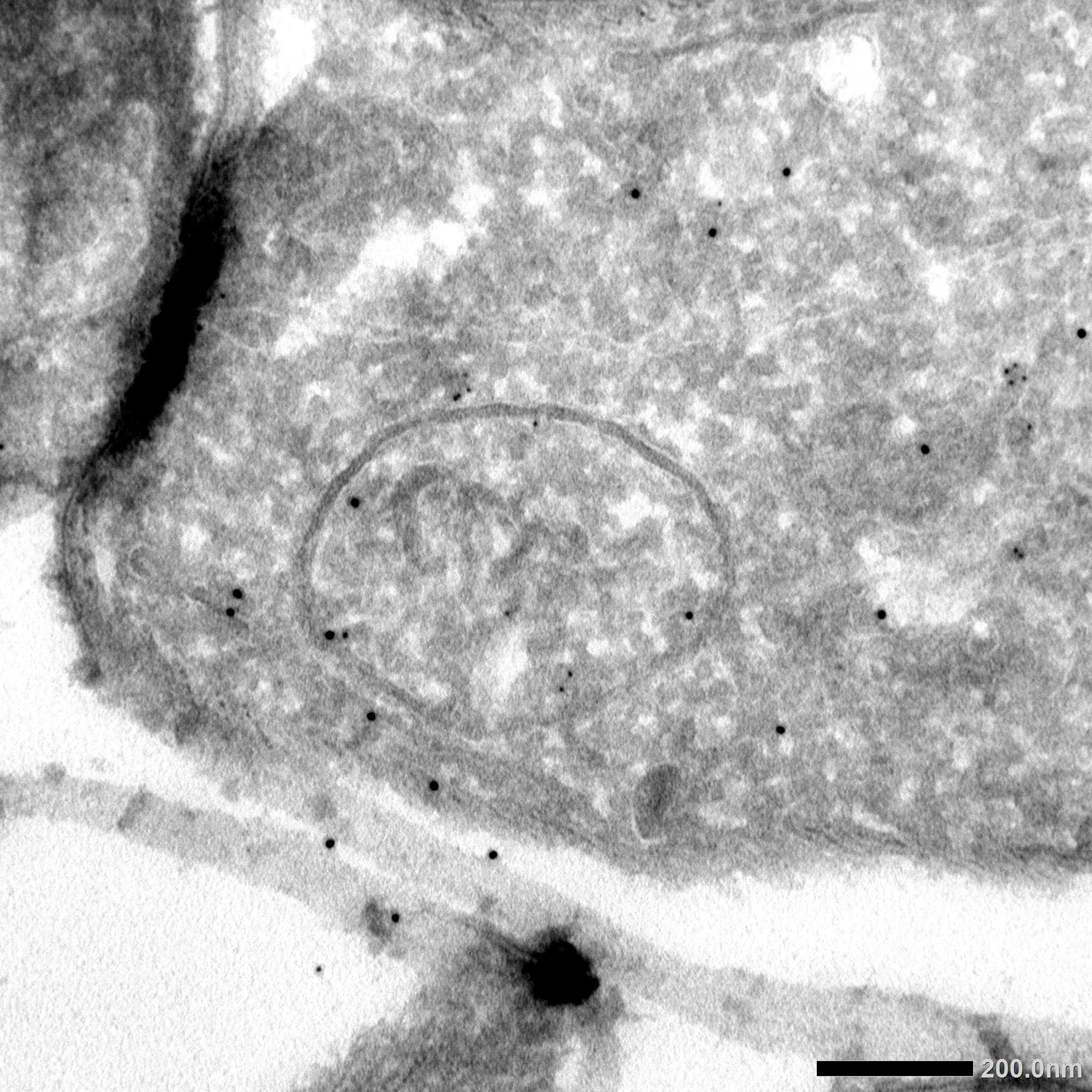

Supplement: Figure 5—source data 1. [file elife-91194-fig5-data1.zip › E.jpg]

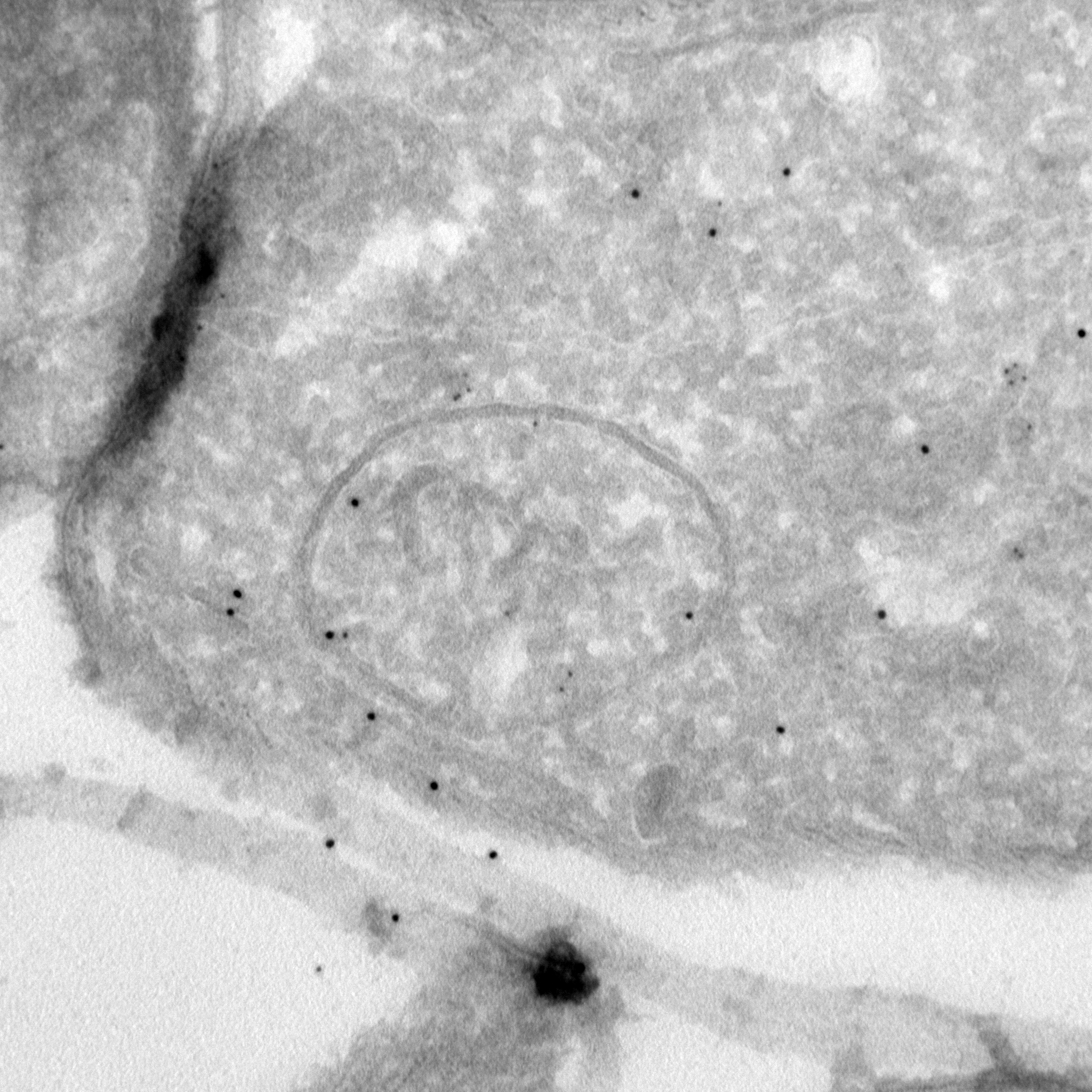

Supplement: Figure 5—source data 1. [file elife-91194-fig5-data1.zip › E.tif]

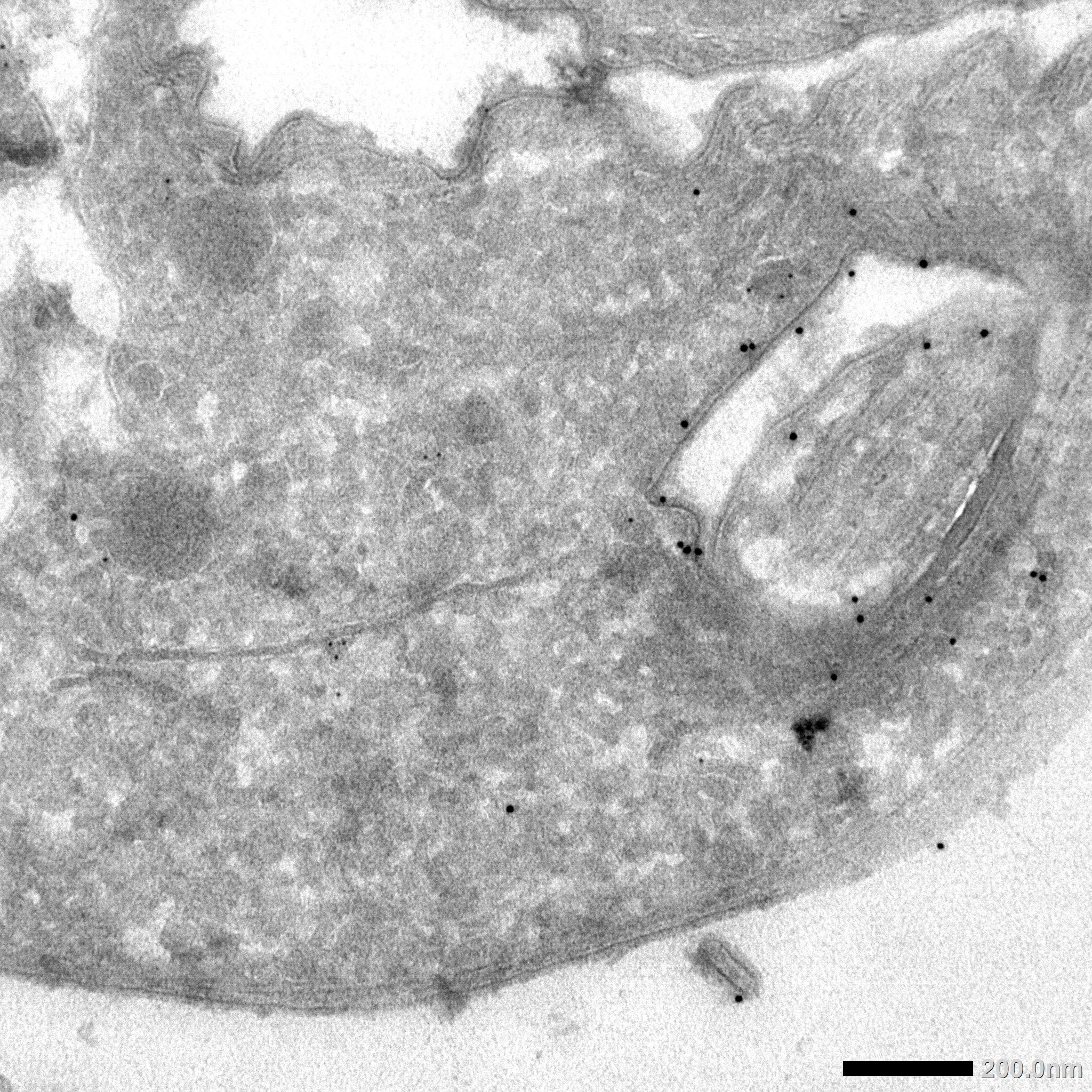

Supplement: Figure 5—source data 1. [file elife-91194-fig5-data1.zip › F.jpg]

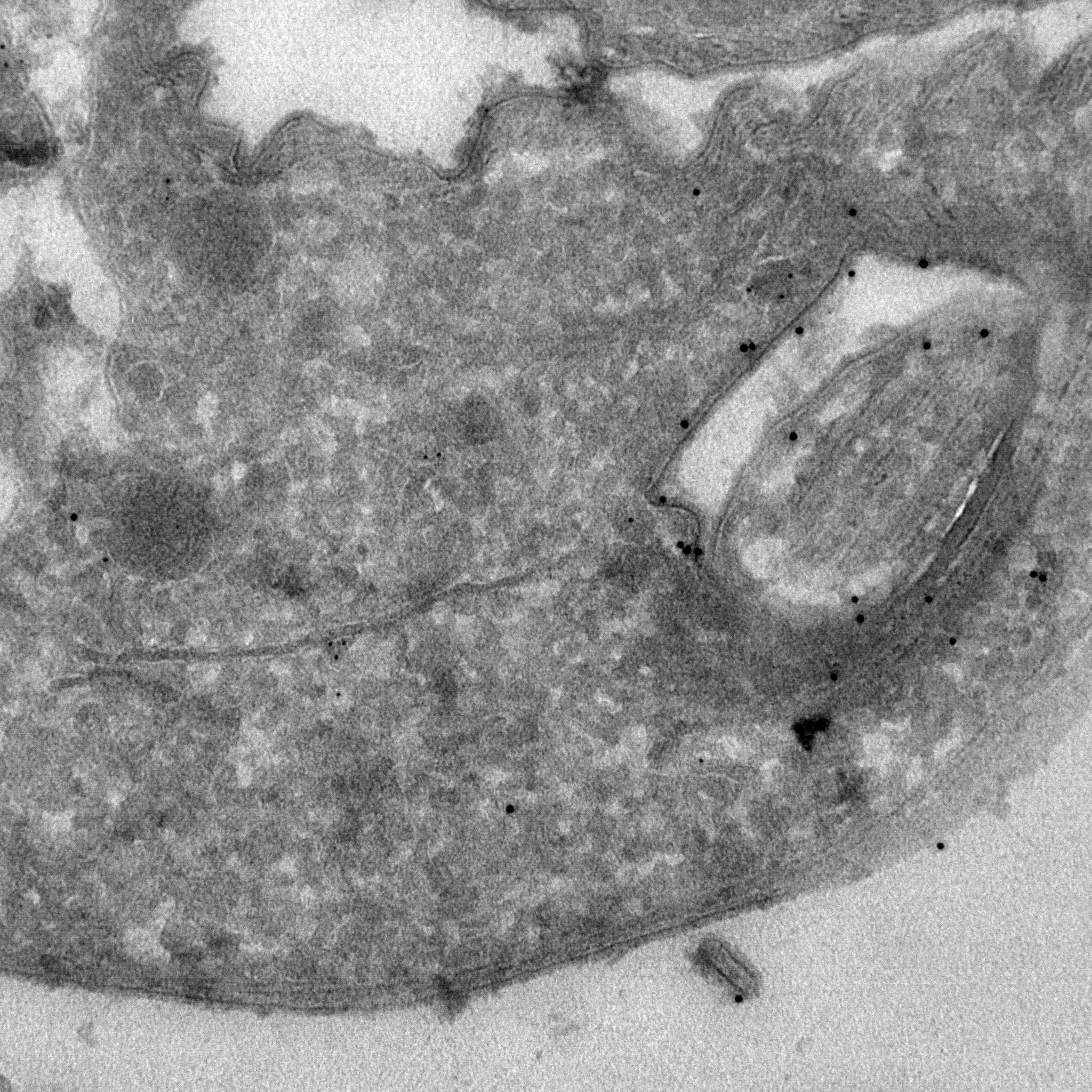

Supplement: Figure 5—source data 1. [file elife-91194-fig5-data1.zip › F.tif]

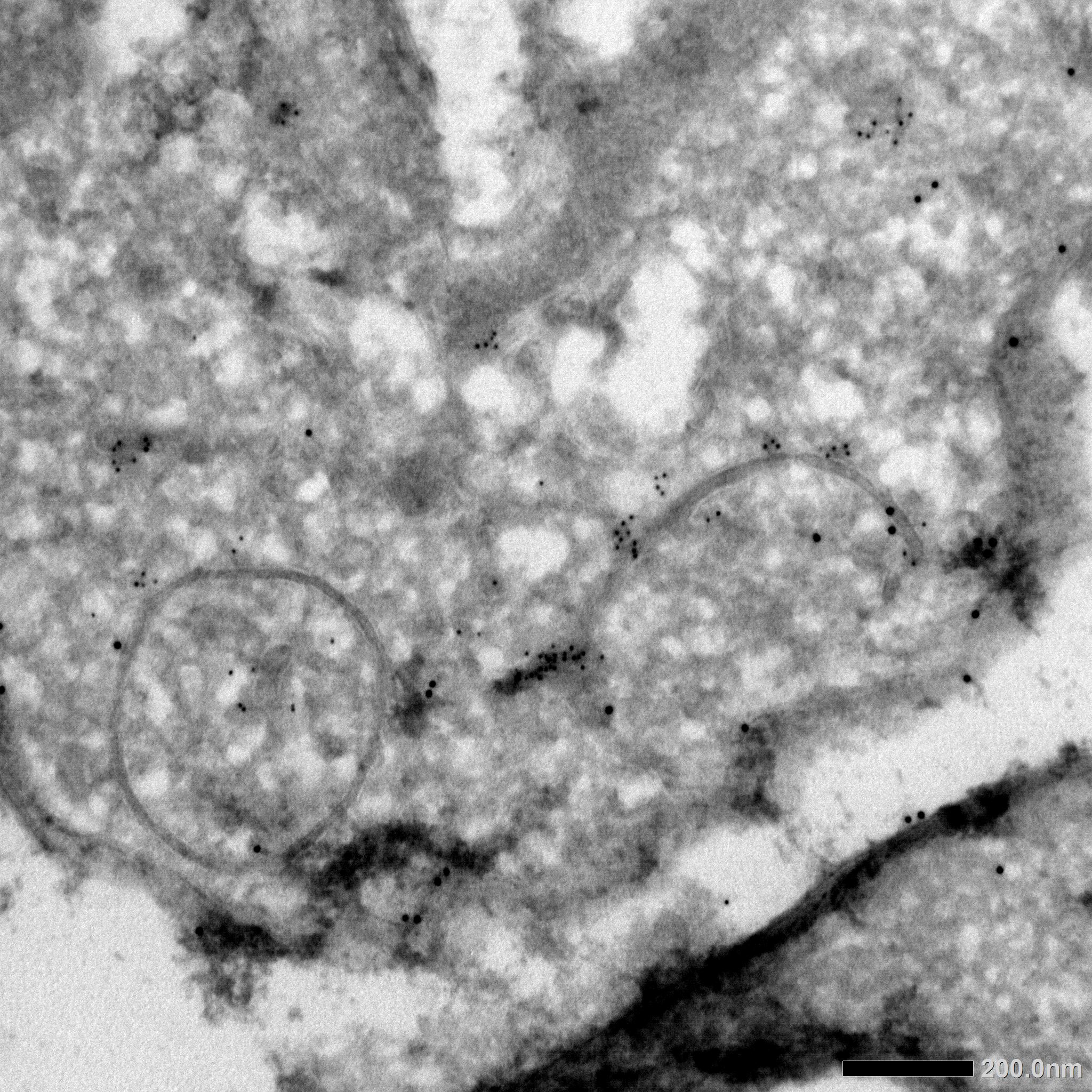

Supplement: Figure 5—source data 1. [file elife-91194-fig5-data1.zip › A, B.jpg]

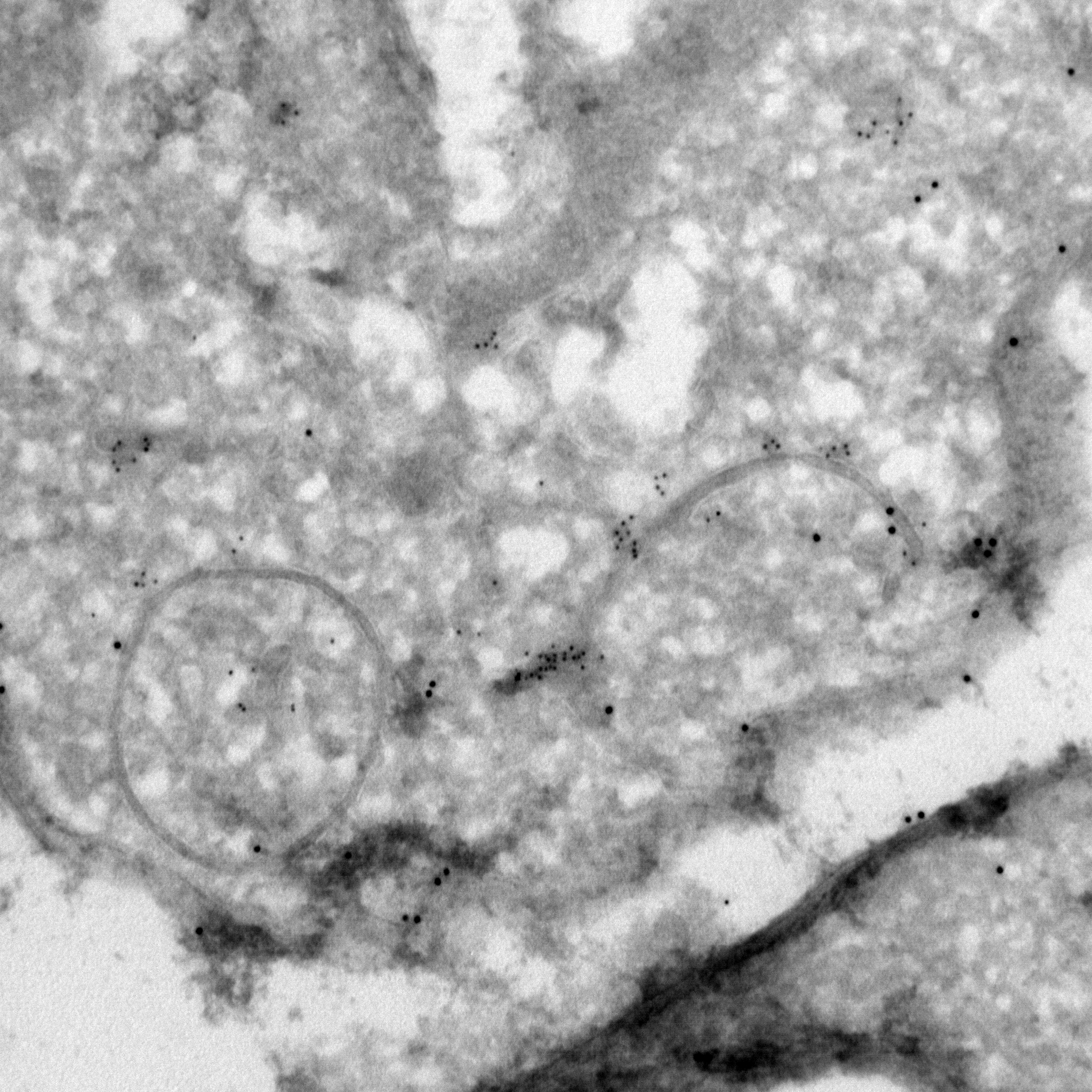

Supplement: Figure 5—source data 1. [file elife-91194-fig5-data1.zip › A, B.tif]

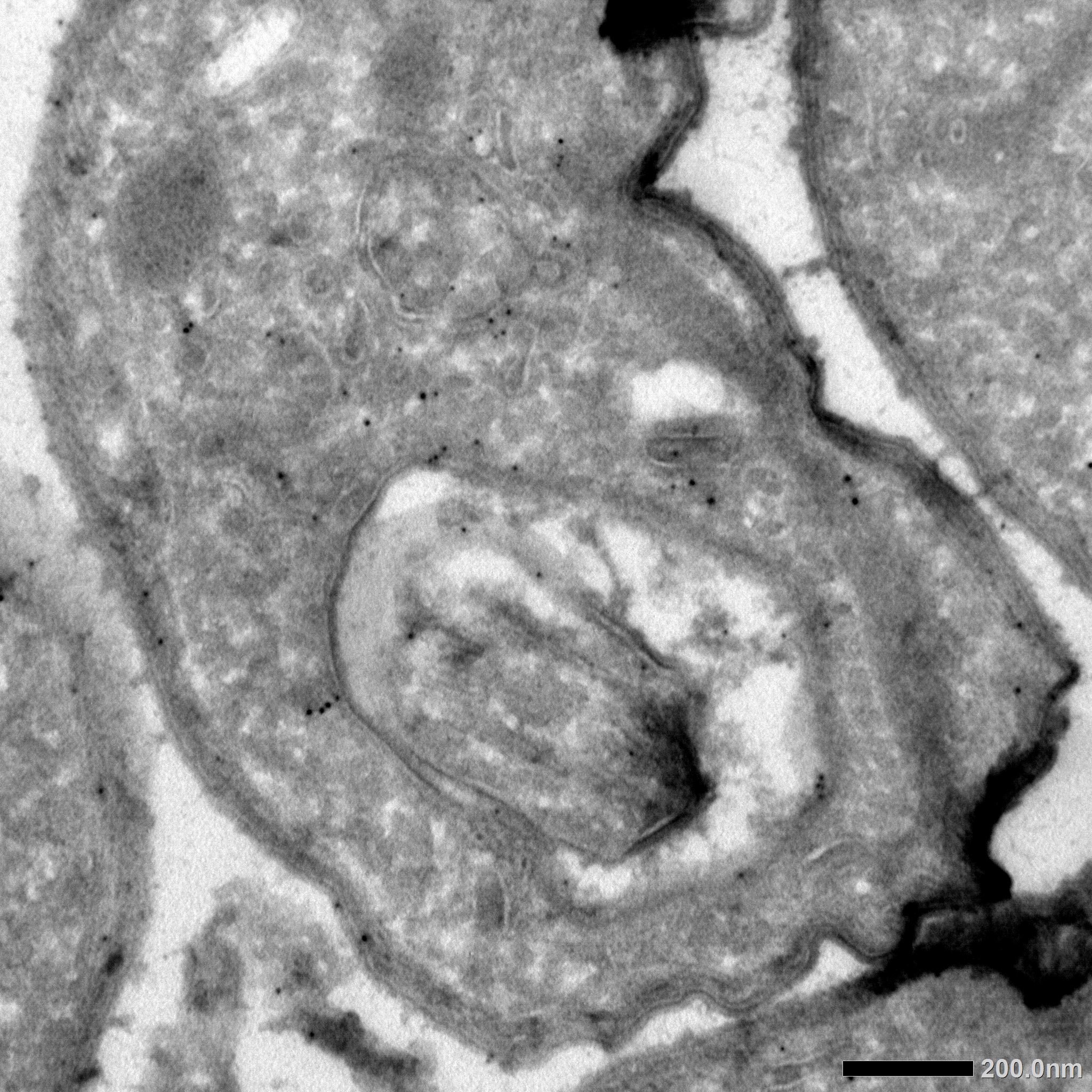

Supplement: Figure 5—source data 1. [file elife-91194-fig5-data1.zip › C.jpg]

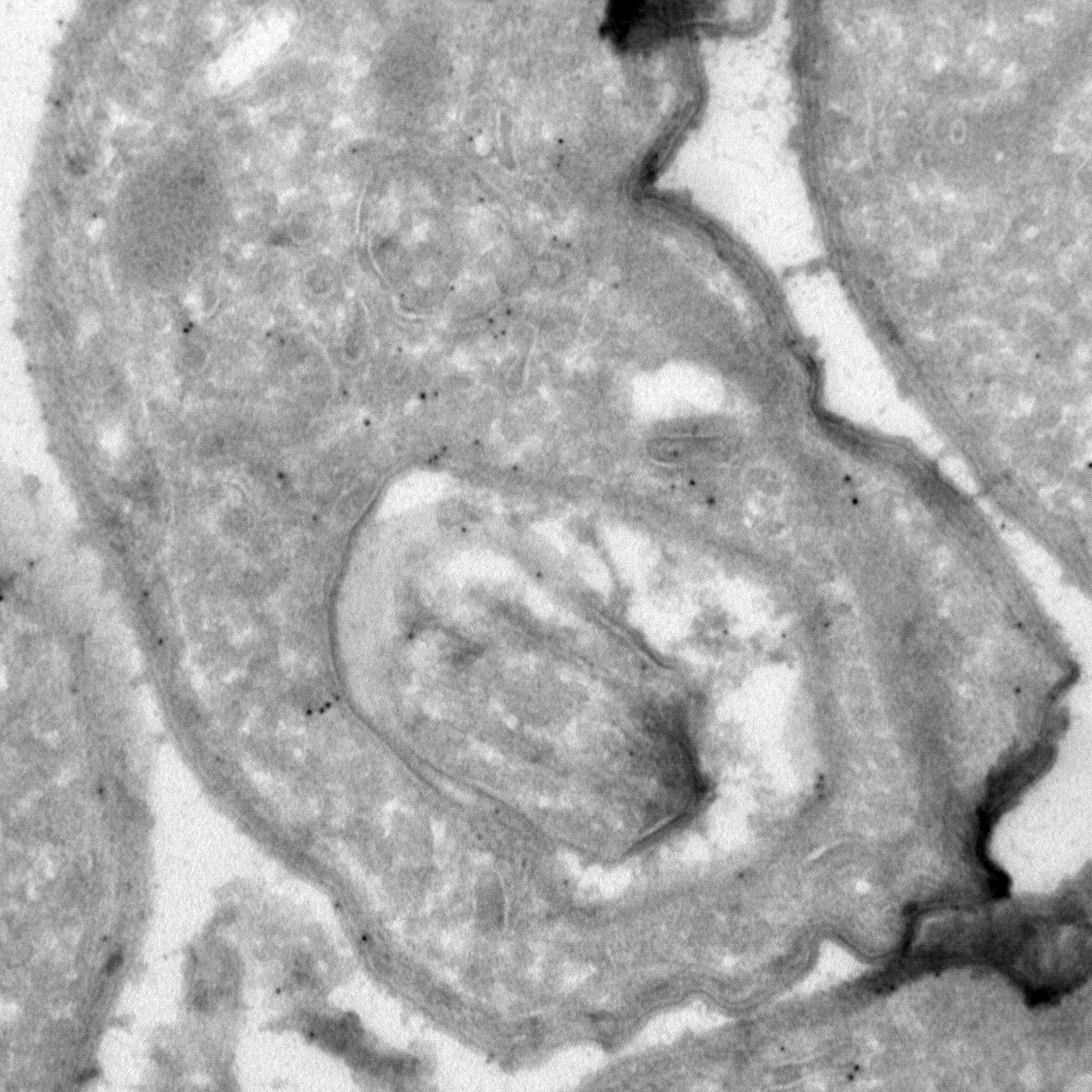

Supplement: Figure 5—source data 1. [file elife-91194-fig5-data1.zip › C.tif]

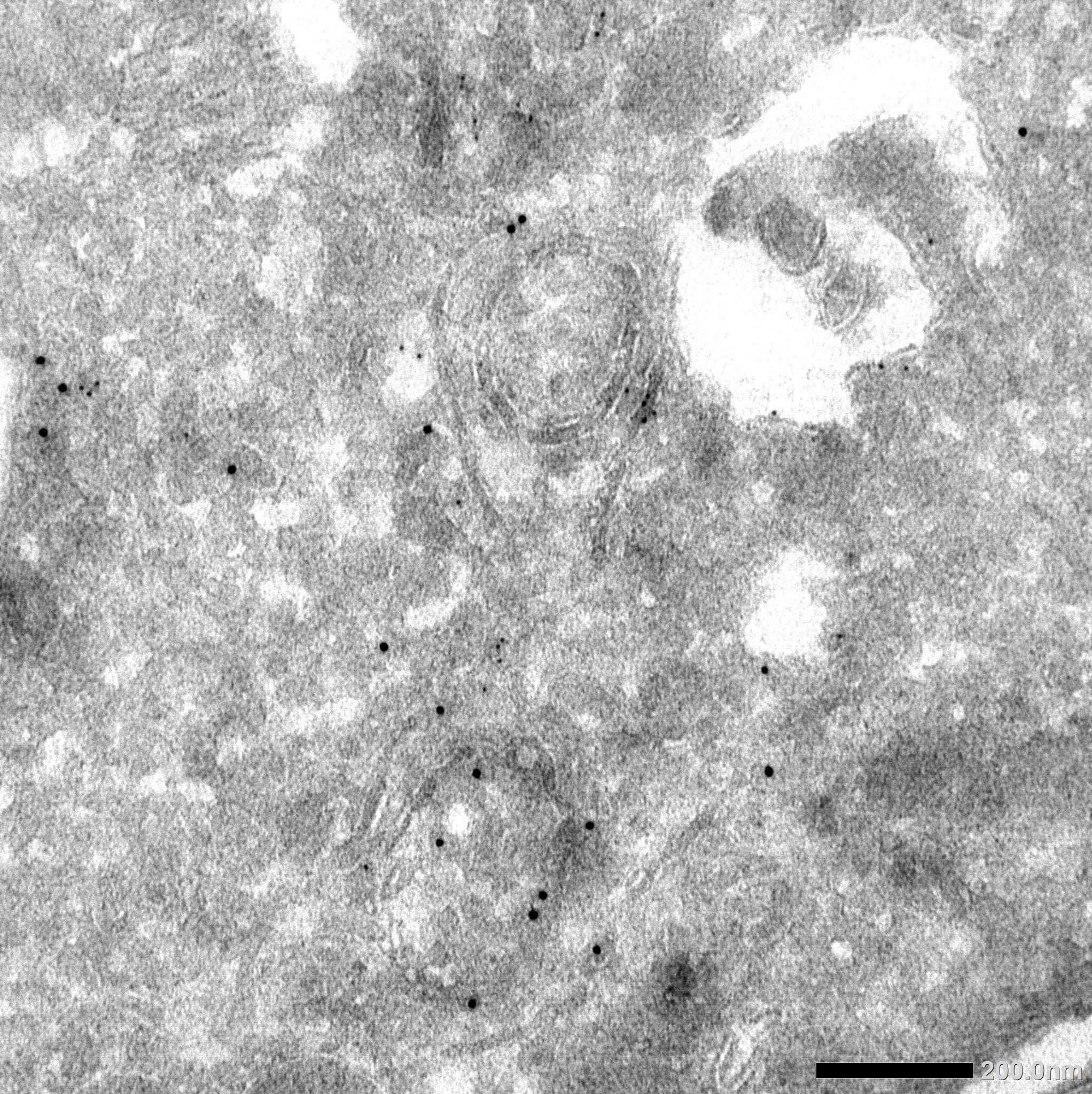

Supplement: Figure 5—source data 1. [file elife-91194-fig5-data1.zip › D.jpg]

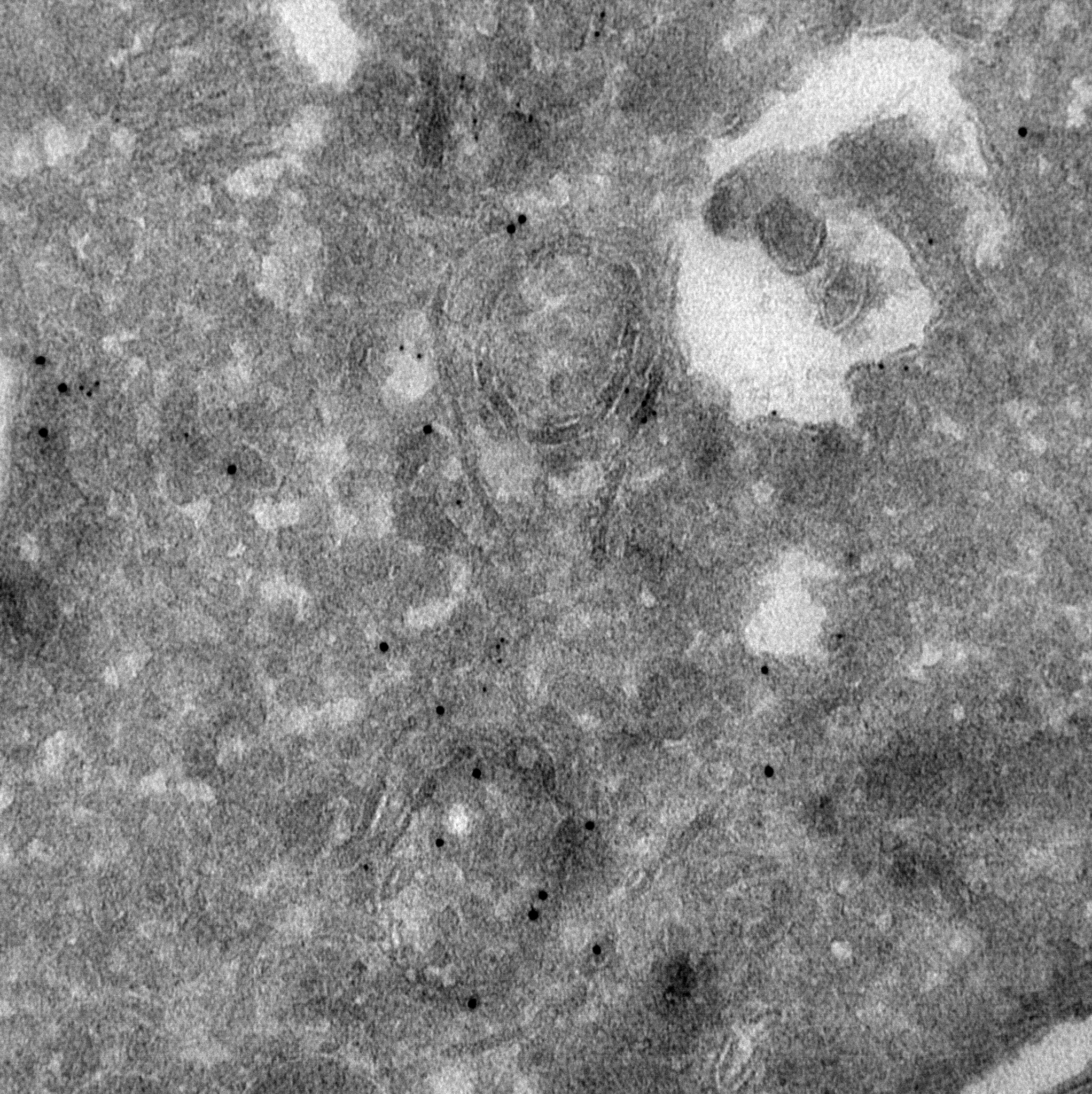

Supplement: Figure 5—source data 1. [file elife-91194-fig5-data1.zip › D.tif]

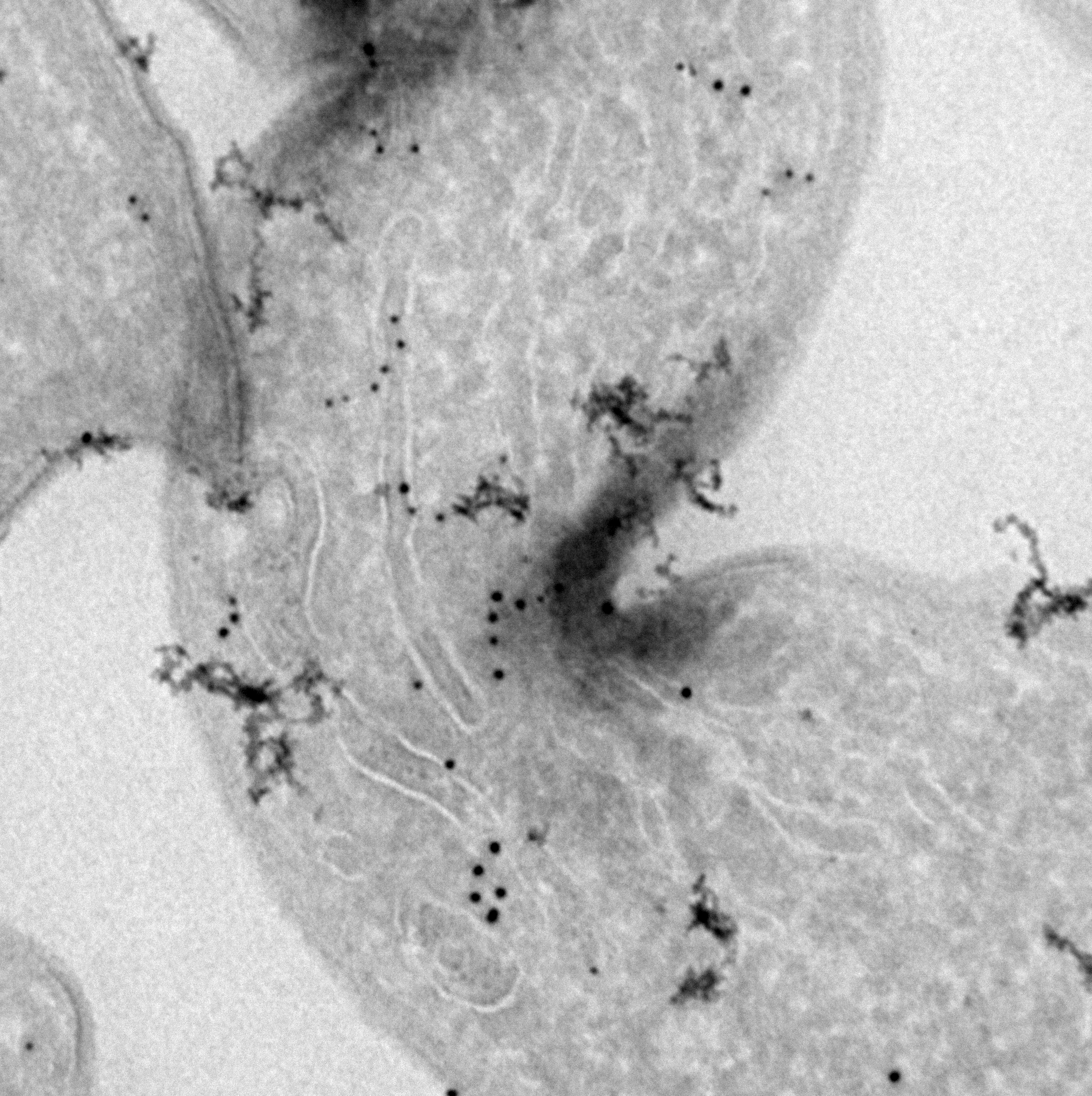

Supplement: Figure 6—source data 1. [file elife-91194-fig6-data1.zip › E.tif]

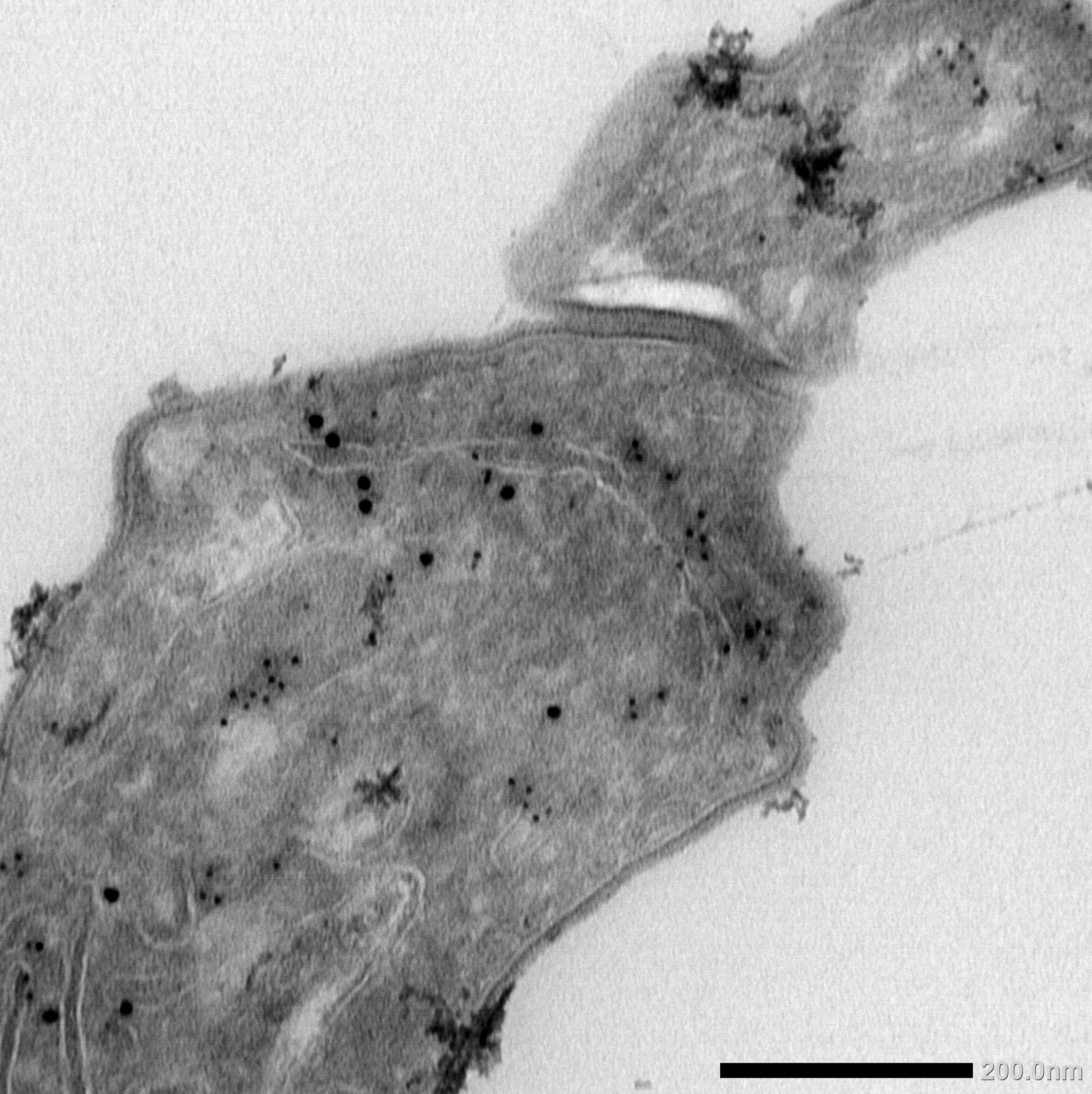

Supplement: Figure 6—source data 1. [file elife-91194-fig6-data1.zip › F.jpg]

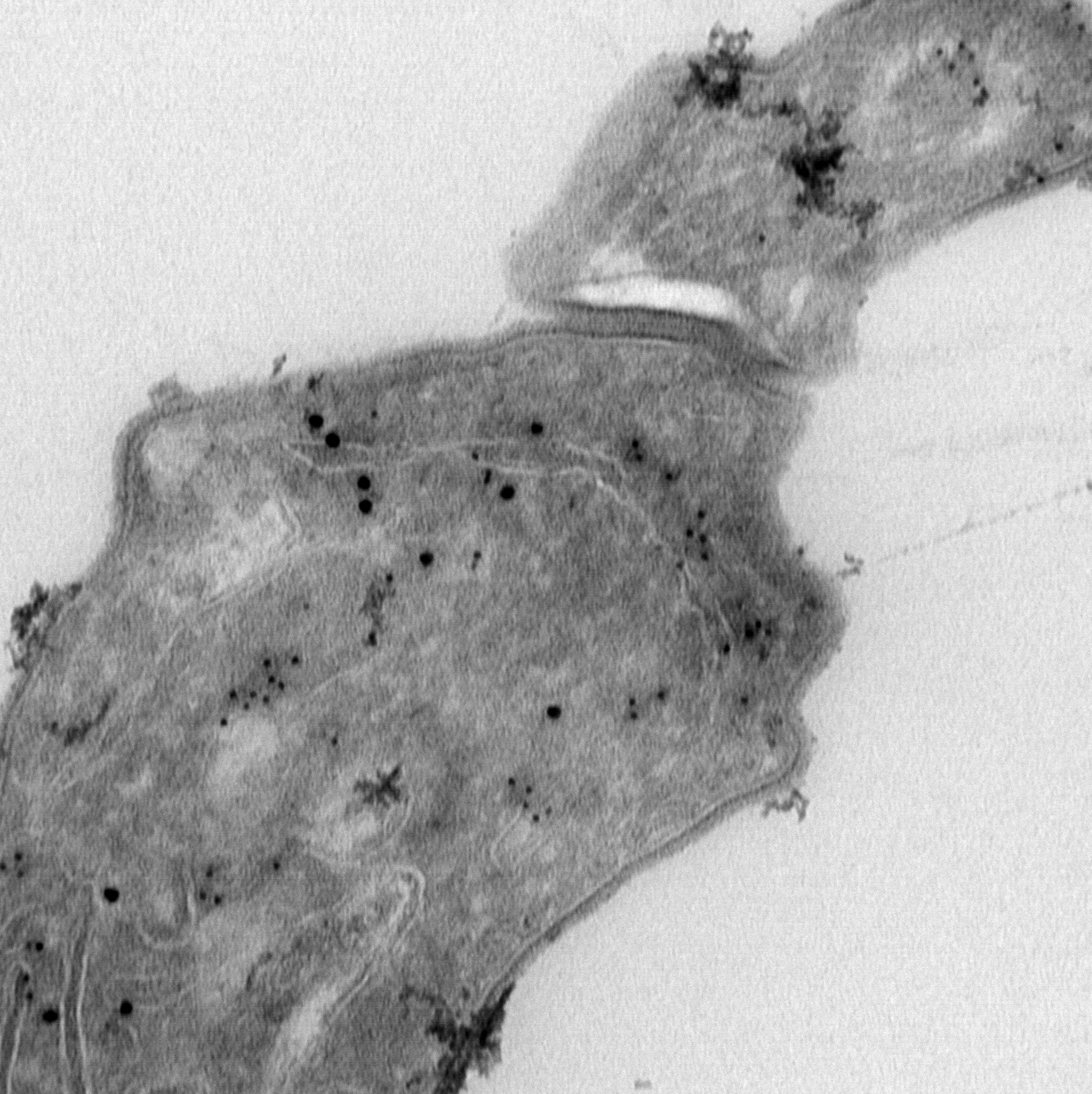

Supplement: Figure 6—source data 1. [file elife-91194-fig6-data1.zip › F.tif]

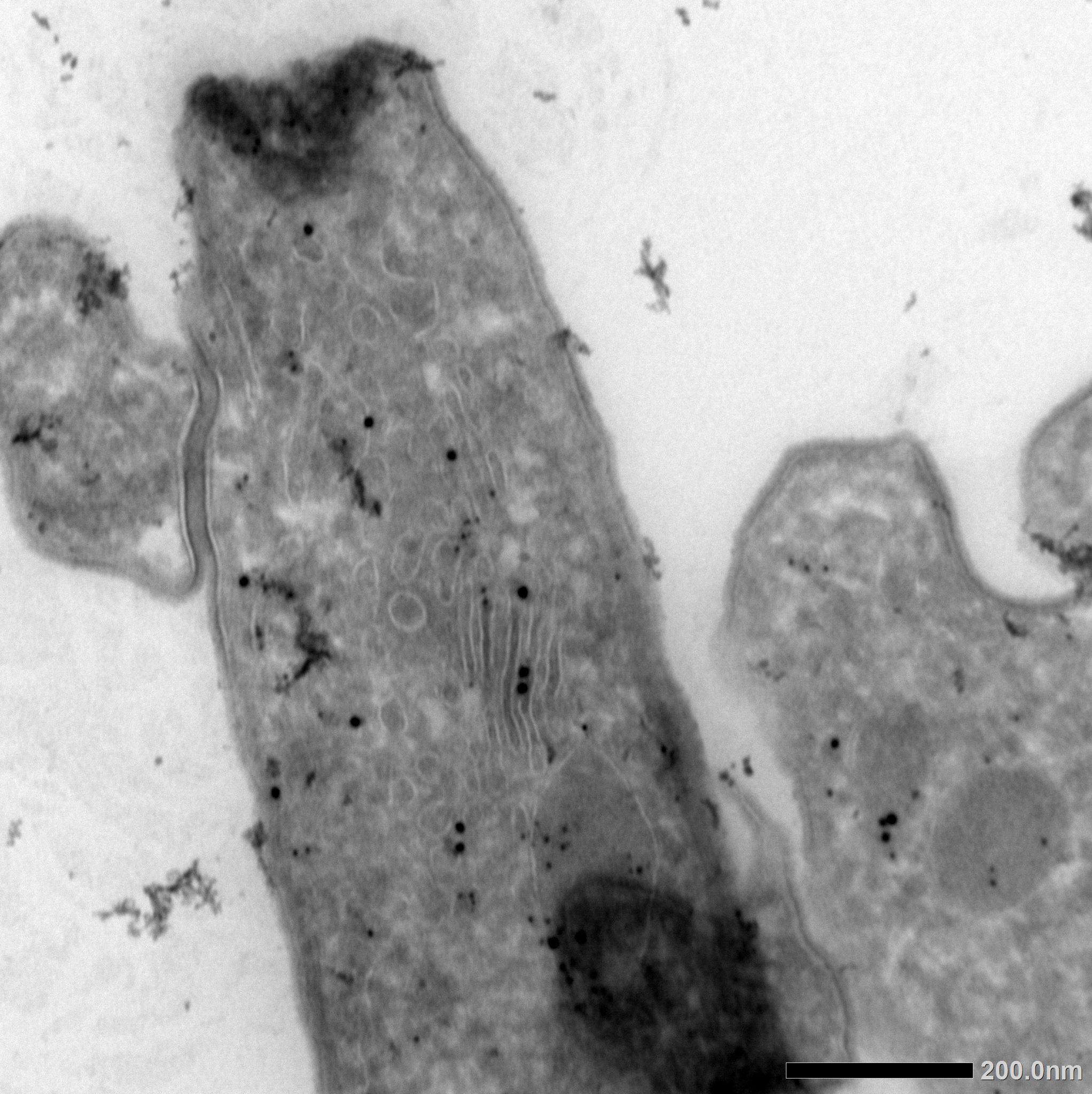

Supplement: Figure 6—source data 1. [file elife-91194-fig6-data1.zip › G.jpg]

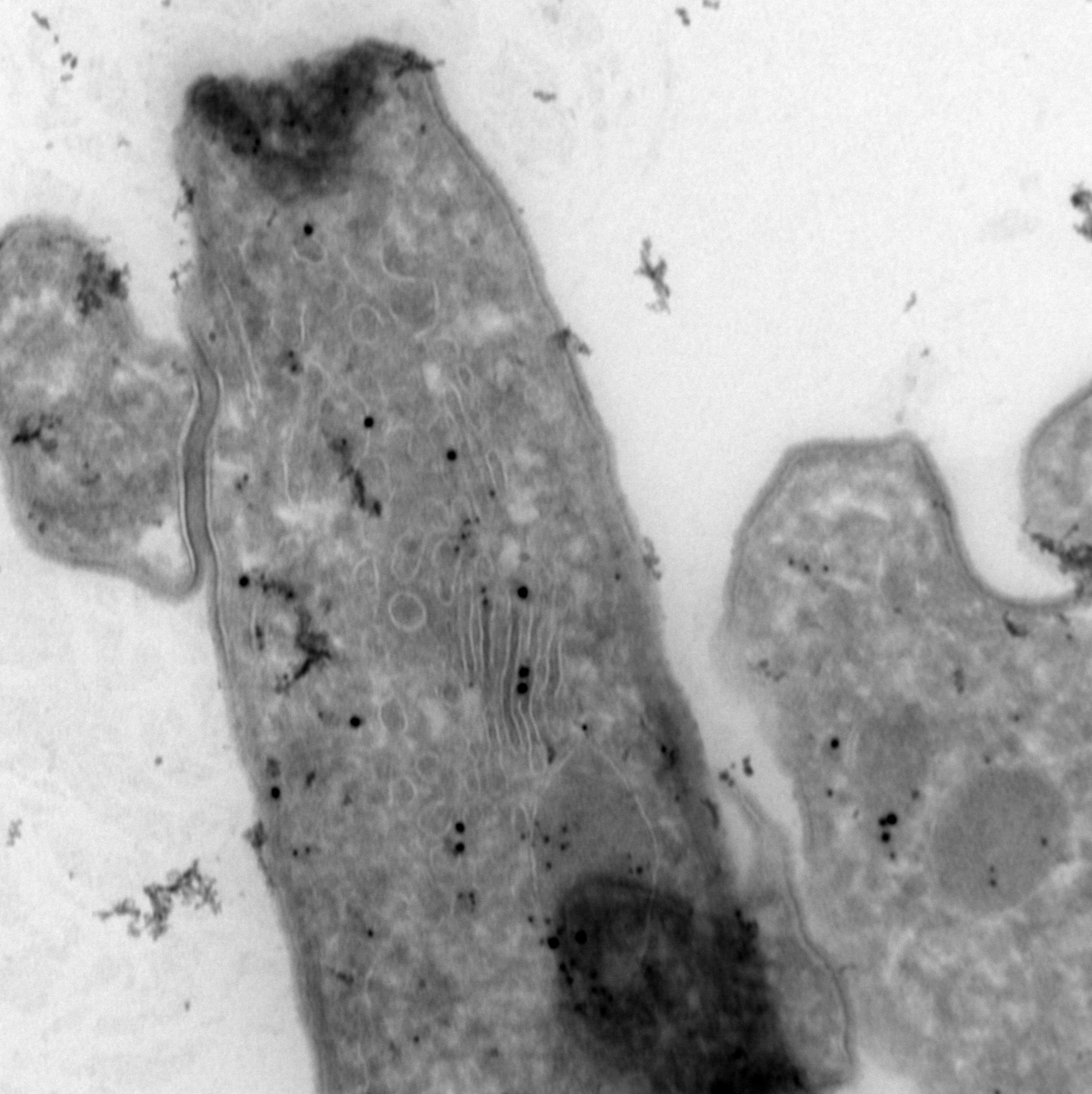

Supplement: Figure 6—source data 1. [file elife-91194-fig6-data1.zip › G.tif]

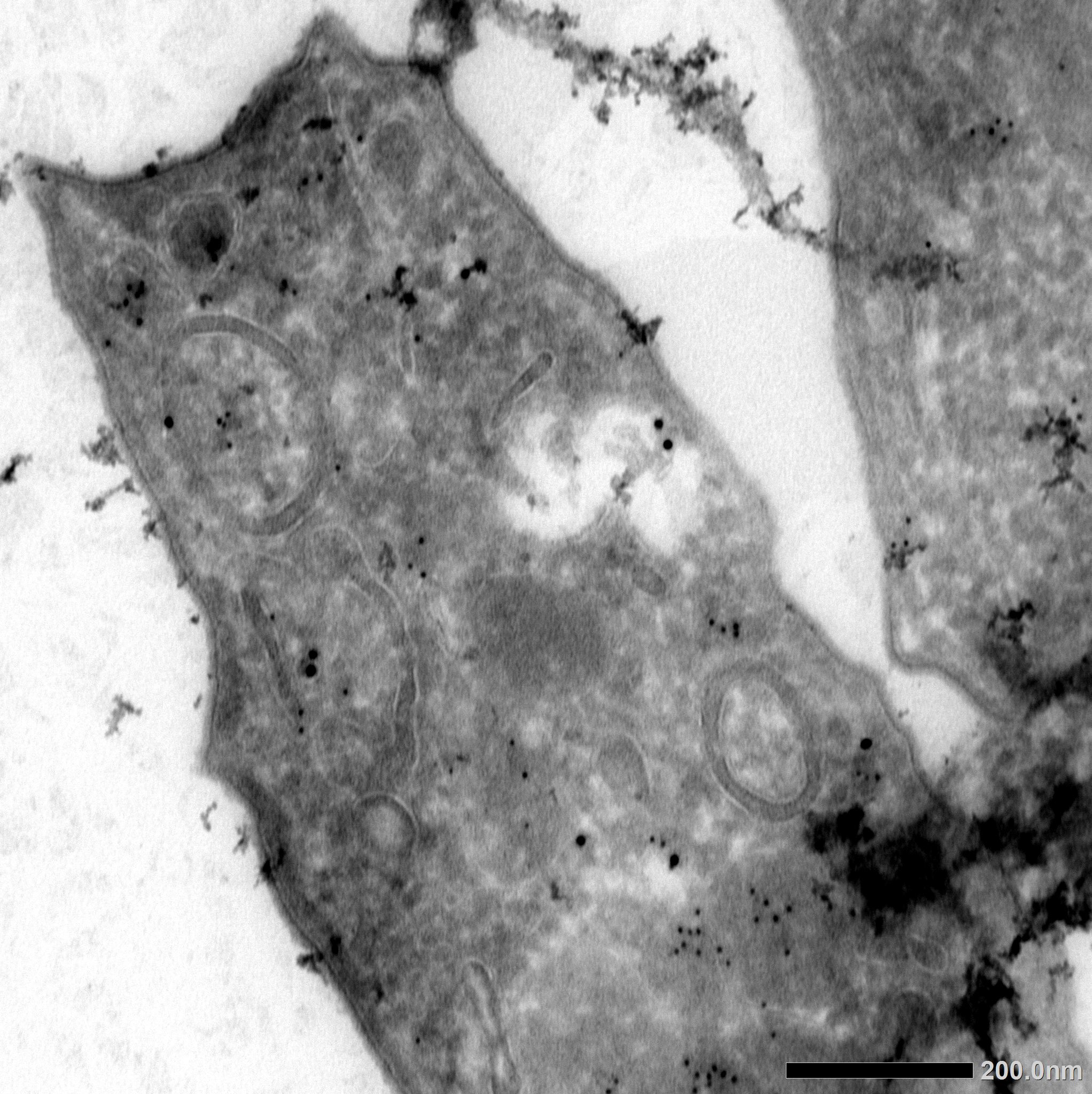

Supplement: Figure 6—source data 1. [file elife-91194-fig6-data1.zip › A.jpg]

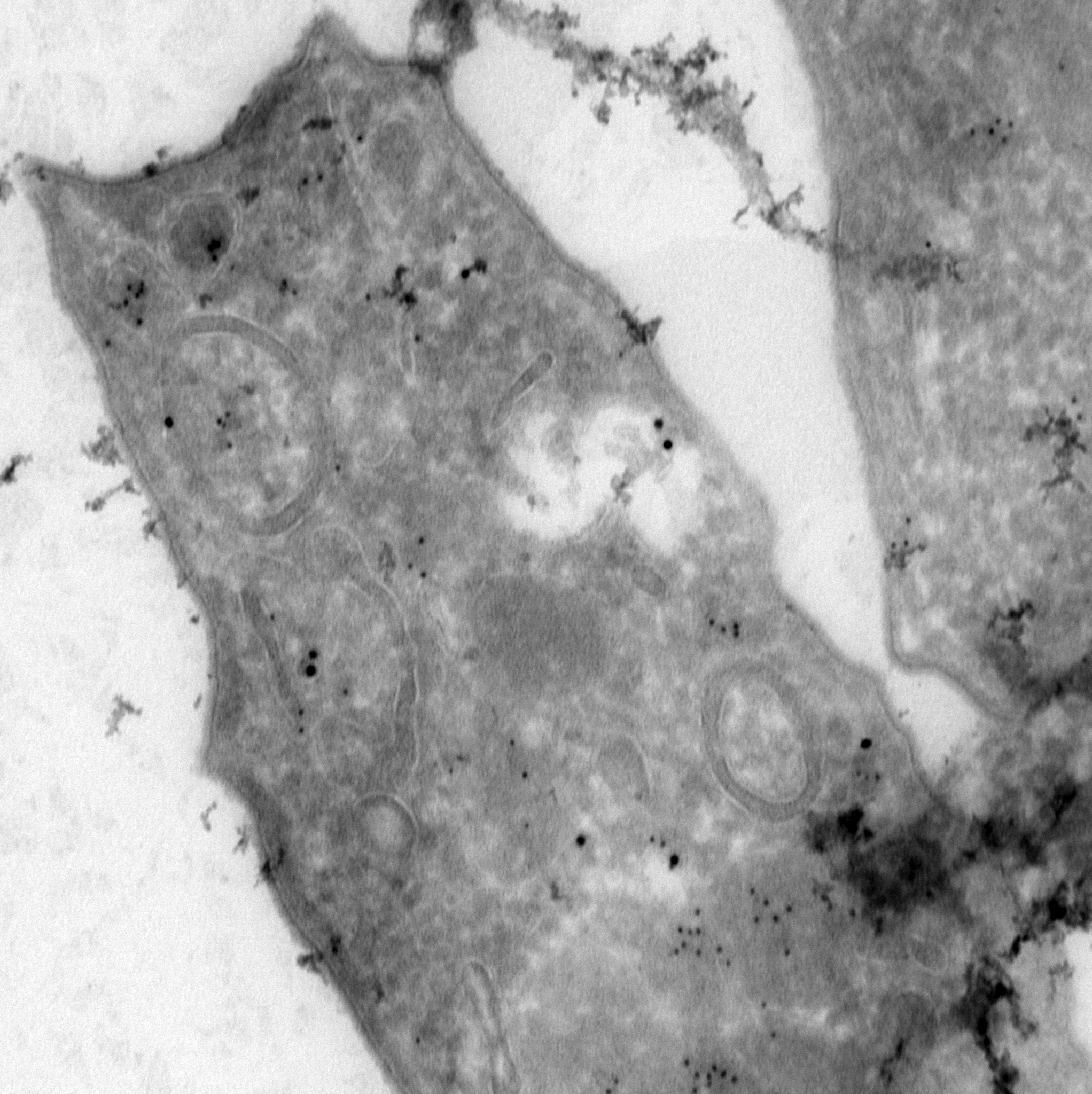

Supplement: Figure 6—source data 1. [file elife-91194-fig6-data1.zip › A.tif]

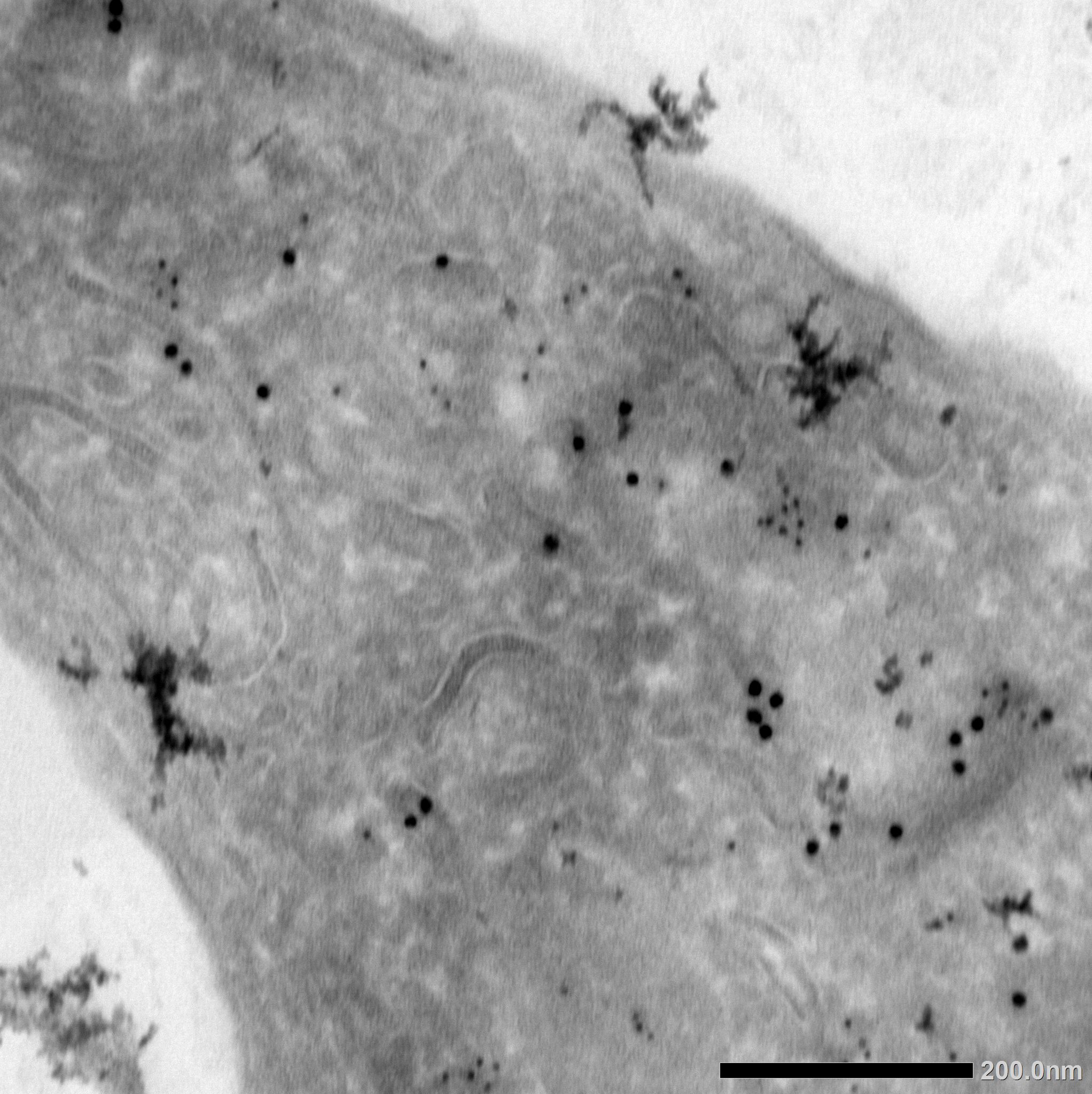

Supplement: Figure 6—source data 1. [file elife-91194-fig6-data1.zip › B.jpg]

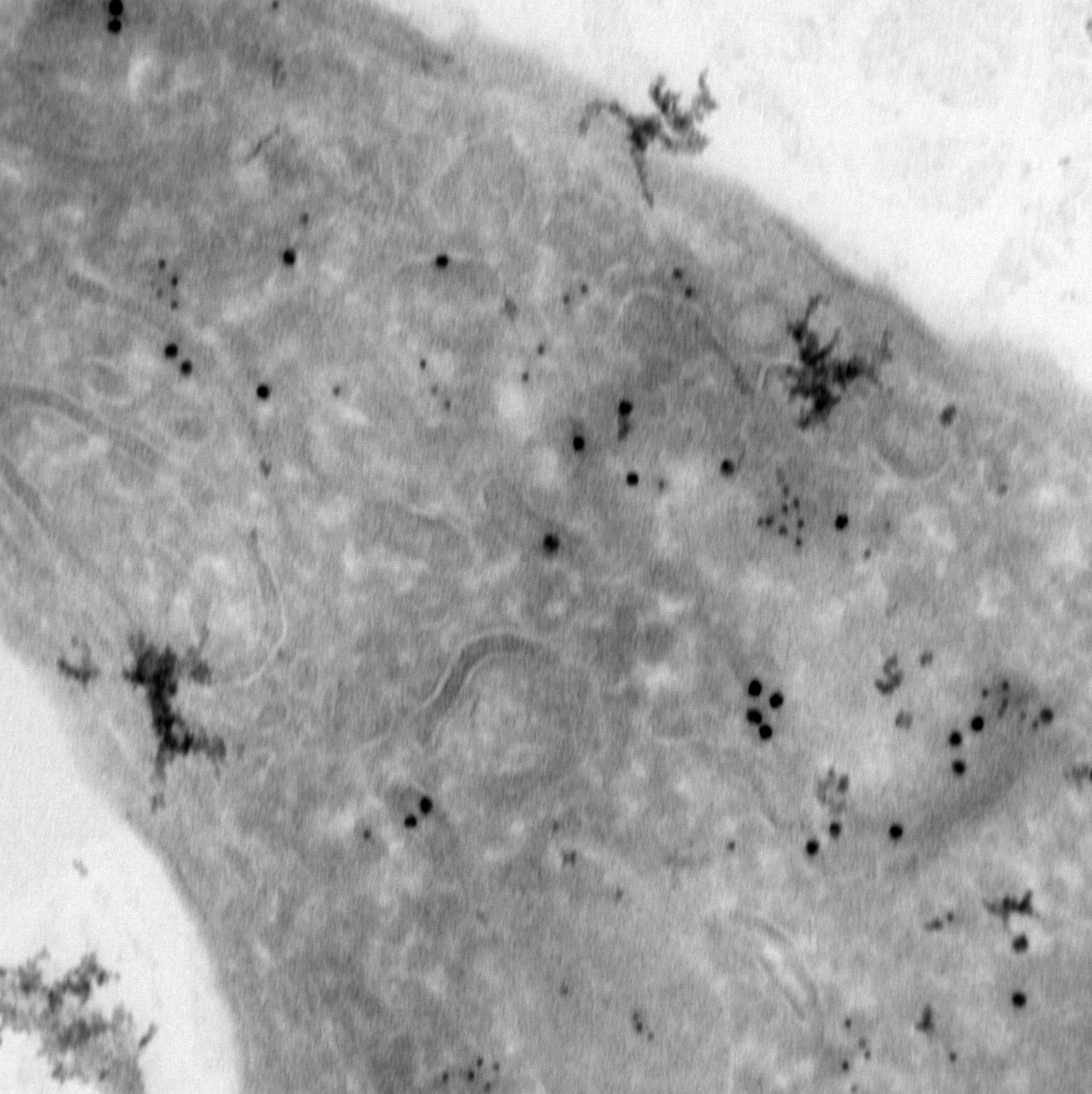

Supplement: Figure 6—source data 1. [file elife-91194-fig6-data1.zip › B.tif]

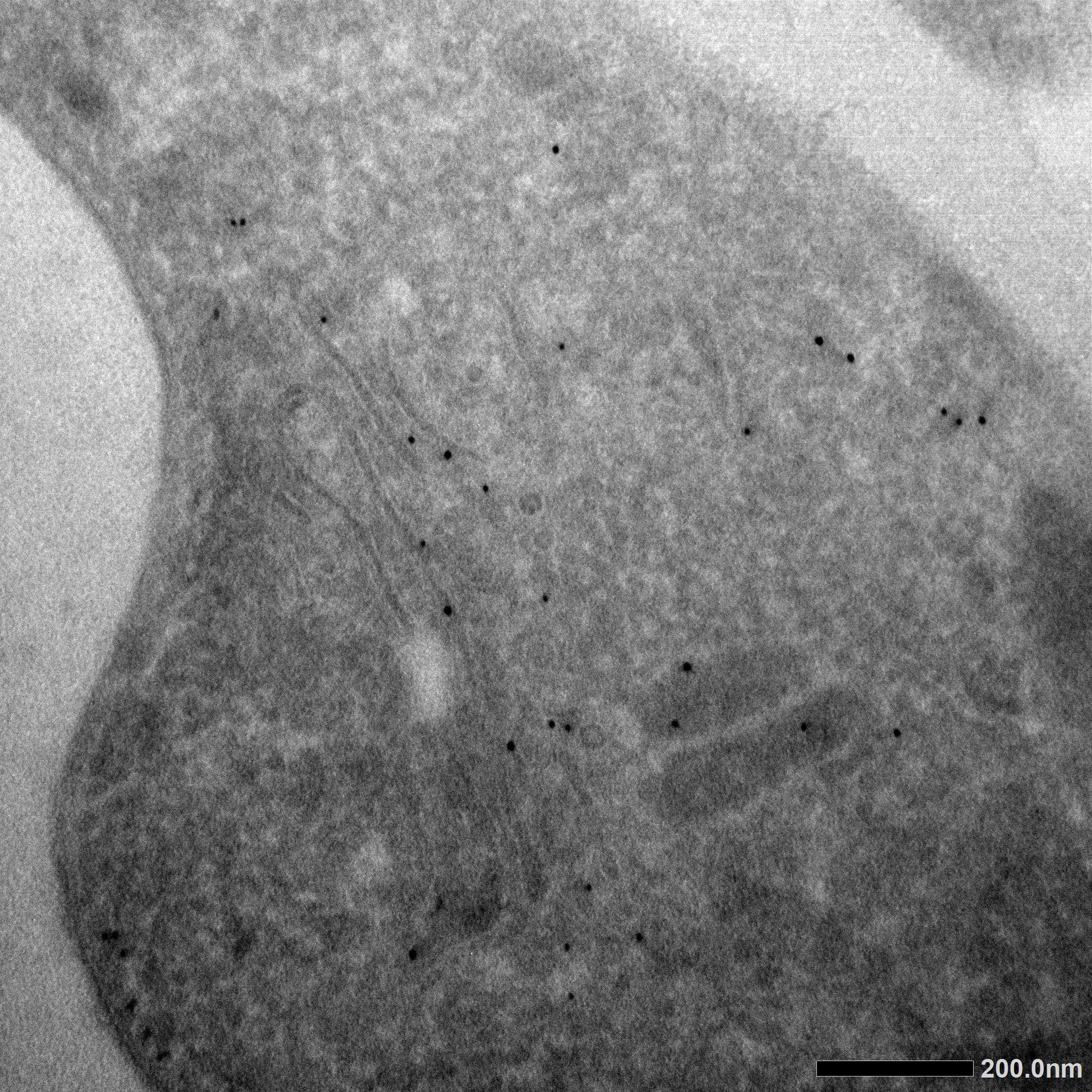

Supplement: Figure 6—source data 1. [file elife-91194-fig6-data1.zip › C.jpg]

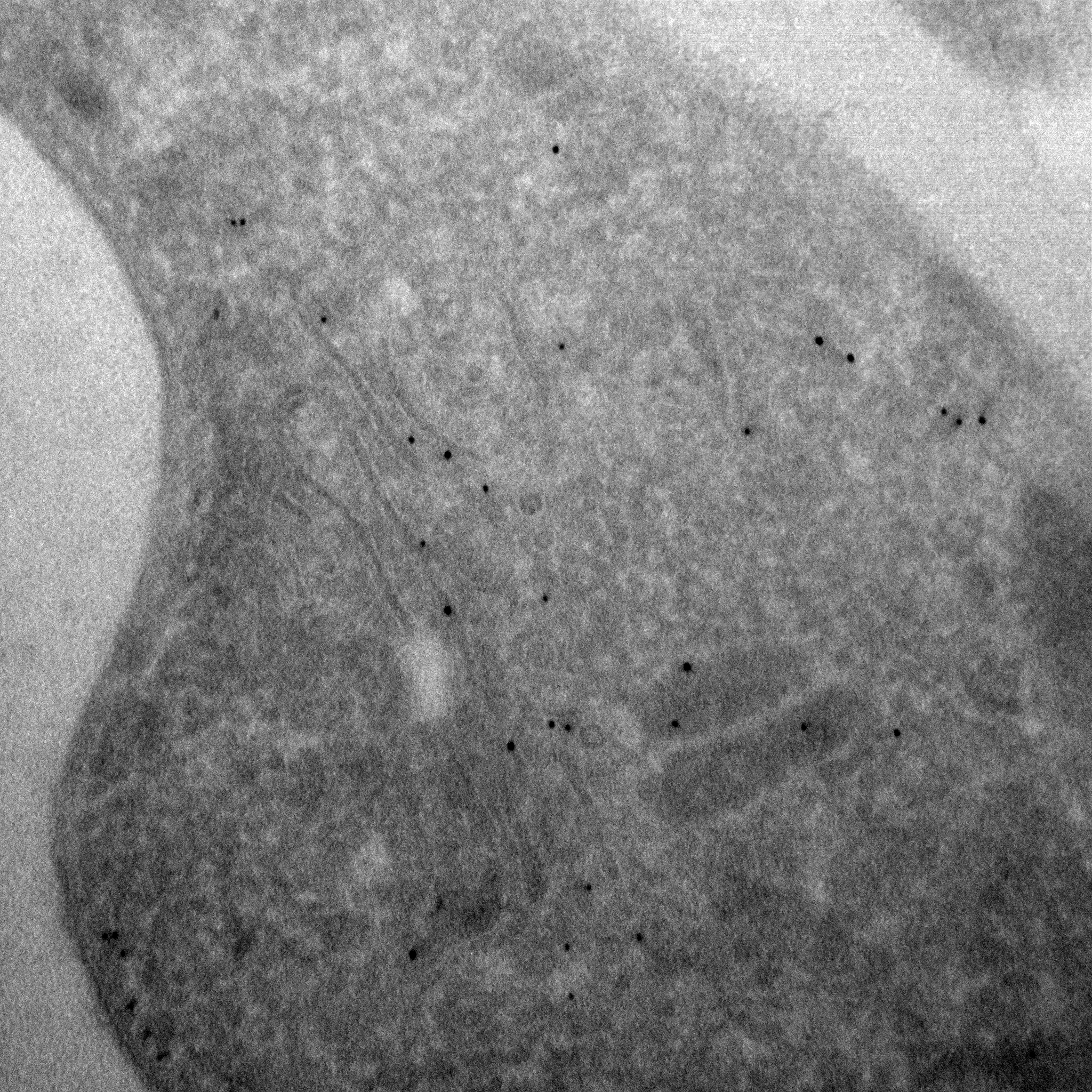

Supplement: Figure 6—source data 1. [file elife-91194-fig6-data1.zip › C.tif]

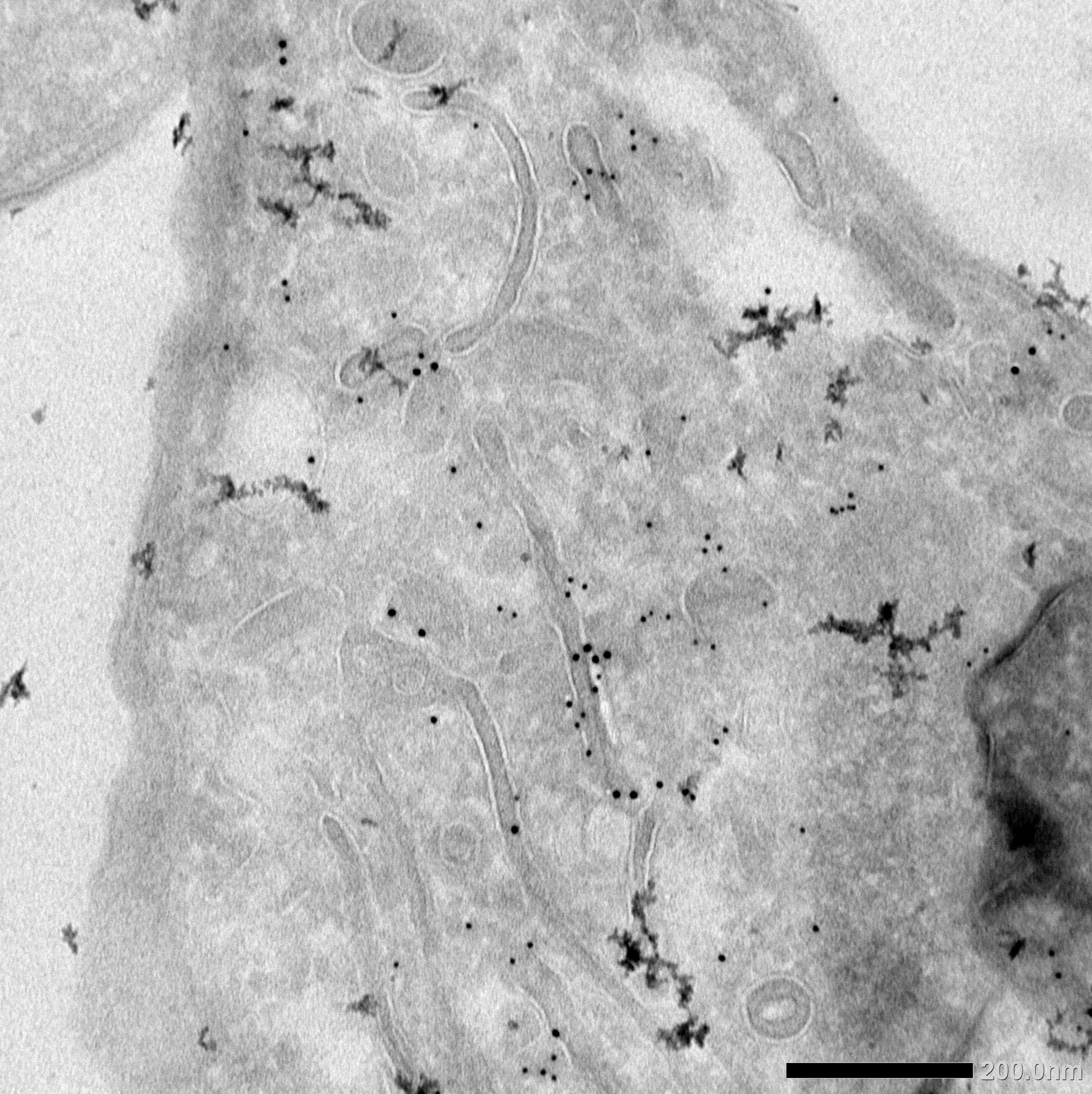

Supplement: Figure 6—source data 1. [file elife-91194-fig6-data1.zip › D.jpg]

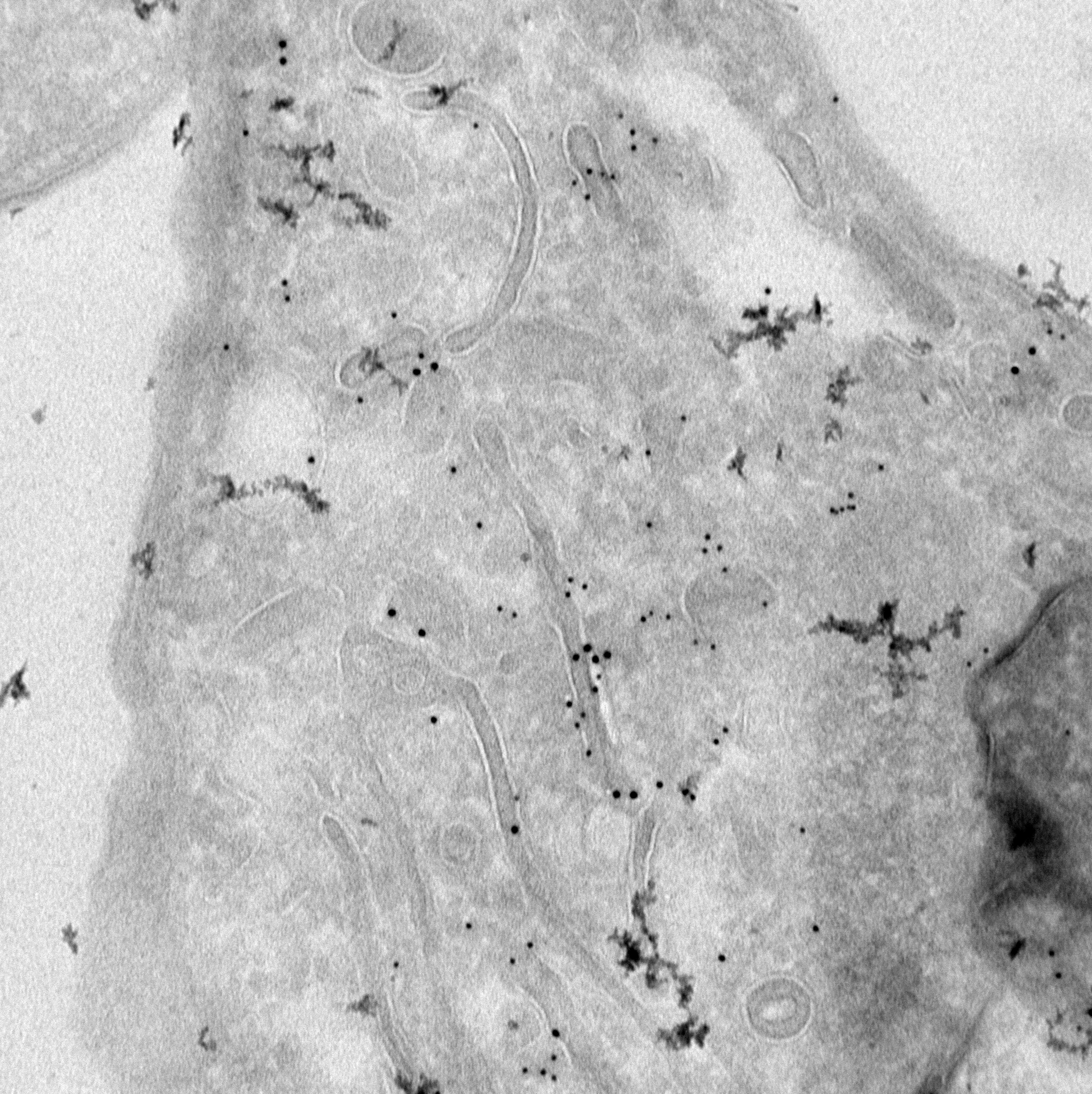

Supplement: Figure 6—source data 1. [file elife-91194-fig6-data1.zip › D.tif]

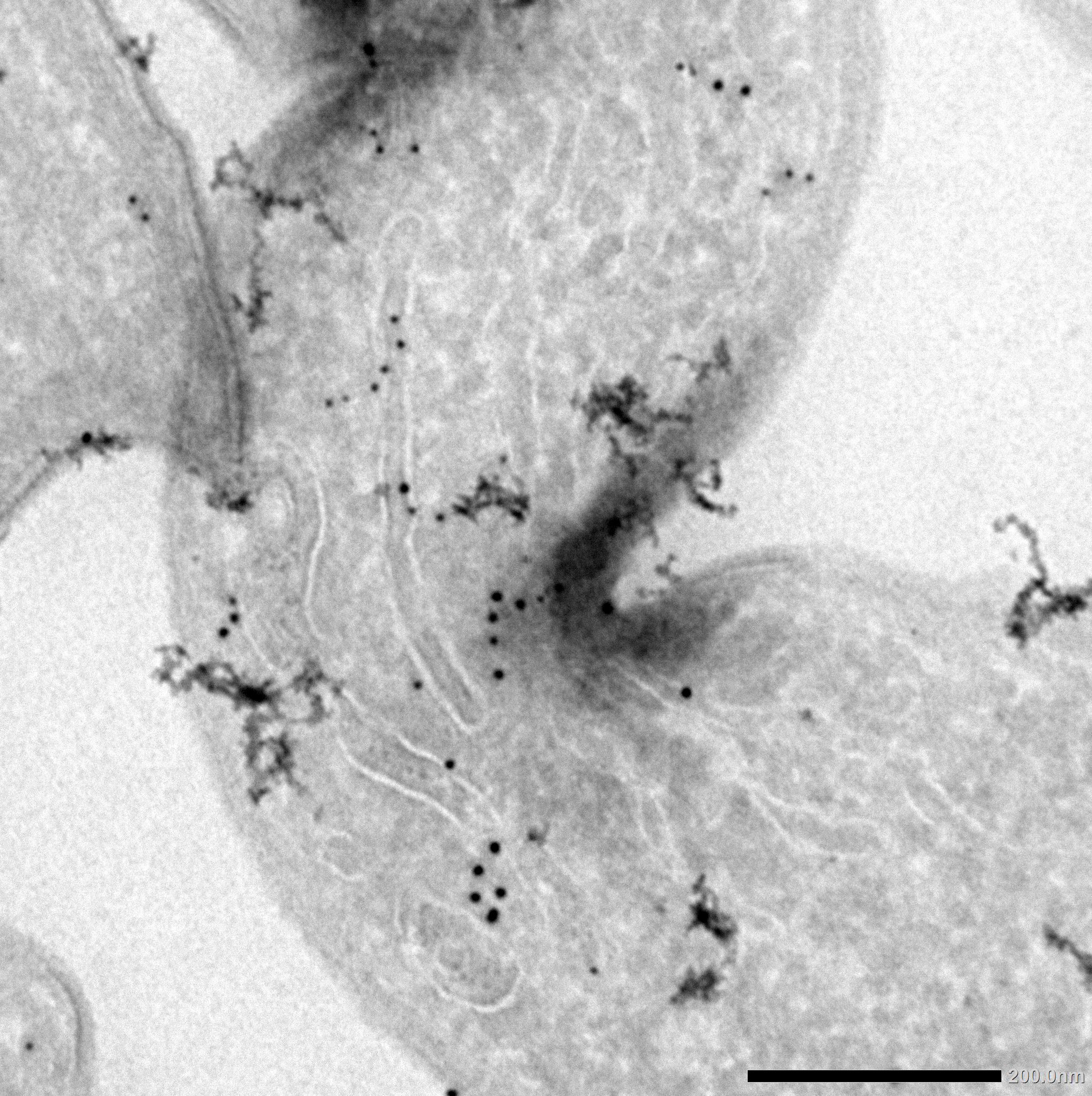

Supplement: Figure 6—source data 1. [file elife-91194-fig6-data1.zip › E.jpg]
